# Inhibition of IRP2-dependent reprogramming of iron metabolism suppresses tumor growth in colorectal cancer

**DOI:** 10.1186/s12964-024-01769-6

**Published:** 2024-08-23

**Authors:** Jieon Hwang, Areum Park, Chinwoo Kim, Chang Gon Kim, Jaesung Kwak, Byungil Kim, Hyunjin Shin, Minhee Ku, Jaemoon Yang, Ayoung Baek, Jiwon Choi, Hocheol Lim, Kyoung Tai No, Xianghua Zhao, Uyeong Choi, Tae Il Kim, Kyu-Sung Jeong, Hyuk Lee, Sang Joon Shin

**Affiliations:** 1 Department of Medicine, Yonsei University College of Medicine, Seoul, 03722 Korea; 2 Songdang Institute for Cancer Research, Yonsei University College of Medicine, Seoul, 03722 Korea; 3 Division of Medical Oncology, Department of Internal Medicine, Yonsei Cancer Center, Yonsei University College of Medicine, Seoul, 03722 Korea; 4 Infectious Diseases Therapeutic Research Center, Korea Research Institute of Chemical Technology, Daejeon, 34114 Korea; 5 Department of Chemistry, Yonsei University, Seoul, 03722 Korea; 6 Division of Gastroenterology, Department of Internal Medicine, Yonsei University College of Medicine, Seoul, 03722 Korea; 7 Department of Radiology, Yonsei University College of Medicine, Seoul, 03722 Korea; 8 Convergence Research Center for Systems Molecular Radiological Science, Yonsei University, Seoul, 03722 Korea; 9 Bioinformatics and Molecular Design Research Center, Incheon, 21983 Korea; 10 The Interdisciplinary Graduate Program in Integrative Biotechnology & Translational Medicine, Yonsei Unversity, Incheon, 21983 Korea; 11 College of Pharmacy, Dongduk Women’s University, Seoul, 02748 Korea

**Keywords:** Iron regulatory protein 2, Iron metabolism, Autophagy, Colorectal cancer

## Abstract

**Background:**

Dysregulation of iron metabolism is implicated in malignant transformation, cancer progression, and therapeutic resistance. Here, we demonstrate that iron regulatory protein 2 (IRP2) preferentially regulates iron metabolism and promotes tumor growth in colorectal cancer (CRC).

**Methods:**

IRP2 knockdown and knockout cells were generated using RNA interference and clustered regularly interspaced short palindromic repeats (CRISPR)-Cas9 methodologies, respectively. Cell viability was evaluated using both CCK-8 assay and cell counting techniques. Furthermore, IRP2 inhibition was determined by surface plasmon resonance (SPR) and RNA immunoprecipitation (IP). The suppressive effects of IRP2 were also corroborated in both organoid and mouse xenograft models, providing a comprehensive validation of IRP2’s role.

**Results:**

We have elucidated the role of IRP2 as a preferential regulator of iron metabolism, actively promoting tumorigenesis within CRC. Elevated levels of IRP2 expression in patient samples are correlated with diminished overall survival, thereby reinforcing its potential role as a prognostic biomarker. The functional suppression of IRP2 resulted in a pronounced delay in tumor growth. Building on this proof of concept, we have developed IRP2 inhibitors that significantly reduce IRP2 expression and hinder its interaction with iron-responsive elements in key iron-regulating proteins, such as ferritin heavy chain 1 (FTH1) and transferrin receptor (TFRC), culminating in iron depletion and a marked reduction in CRC cell proliferation. Furthermore, these inhibitors are shown to activate the AMPK-ULK1-Beclin1 signaling cascade, leading to cell death in CRC models.

**Conclusions:**

Collectively, these findings highlight the therapeutic potential of targeting IRP2 to exploit the disruption of iron metabolism in CRC, presenting a strategic advancement in addressing a critical area of unmet clinical need.

**Supplementary Information:**

The online version contains supplementary material available at 10.1186/s12964-024-01769-6.

## Introduction

Iron serves as a fundamental element for DNA synthesis [1, 2], mitochondrial respiration, and cell proliferation [3–5], playing a central role in redox reactions, mitochondrial functionality, and cell cycle regulation [6, 7]. Particularly, dysregulation of iron metabolism is frequently observed in cancer cells, necessitating a higher iron demand for maintaining accelerated growth rates compared to normal cells.

In mammals, iron regulatory proteins 1 and 2 (IRP1 and 2) post-transcriptionally control intracellular iron homeostasis through binding of iron responsive elements (IREs) to the 5′- or 3′-untranslated region (UTR) of the selected mRNAs [8, 9]. In general, the binding of iron regulatory proteins to IREs in the 5′-UTR of mRNA suppresses the translation of proteins such as ferritin heavy chain 1 (FTH1) and ferroportin (FPN) [10, 11], whereas binding to IREs in the 3′-UTR of mRNA confers protein stability via transferrin receptor (TFRC) and divalent metal transporter 1 (DMT1) [12, 13].

Sharing a high degree of nucleotide sequence homology, IRP1 and IRP2 are differentially expressed on the basis of cellular iron availability, and are crucial for iron metabolism reprogramming [7]. IRP1 is a ubiquitously expressed protein and has a 4Fe-4S cluster that hinders the binding of IRP1 to IREs, whereas IRP2 is a selectively expressed protein that has no iron-sulfur cluster and is reported to be the dominant IRE-binding protein [14]. Additionally, the degradation of IRP2 is modulated by F-box and leucine-rich repeat protein 5 (FBXL5), an E3 ubiquitin ligase [15, 16].

Colorectal cancer (CRC) is the second most frequent cause of cancer-related deaths worldwide, following lung cancer [17]. There are markedly few therapeutic drugs specifically developed for CRC treatment. For example, monoclonal antibodies against the epidermal growth factor receptor display an effective response in only approximately half of the patients without tumors harboring mutations in the RAS/RAF/MAPK pathway [18, 19]. Additionally, newly developed immunotherapy is promising but acceptable to only less than 5% of the patients with metastatic CRC with microsatellite instability-high tumors [20]. Despite an increase in the incidence of CRC, progress in the development of anti-cancer drugs for CRC is stagnant and the survival rate of patients in stage IV CRC for over 5 years remains 15% [21]. Although attempts have been made to investigate the importance of iron excess and its direct effect on CRC development and progression [22], methods for reprogramming iron metabolism in cancer cells have been nearly absent. By exploiting the differences in iron metabolism between cancer and normal cells, we hypothesized that targeting IRP2 specifically can inhibit cancer cell proliferation, leading to an effective cancer treatment.

Accordingly, we aimed to determine the significance of IRP2 as a therapeutic target in cancer. Extending this foundational concept, we discovered IRP2 inhibitors through high-throughput screening of a library of 8.0 million compounds. We found that genetic ablation or pharmacologic inhibition of IRP2 disrupts cellular iron homeostasis and represses colon cancer cell growth in diverse model systems, including patient-derived cell lines, organoids, and tumor xenografts, thereby highlighting the feasibility of targeting IRP2 in CRC.

## Results

### Inhibition of IRP2 impairs cell growth in colorectal cancer (CRC)

**Fig. 1:**
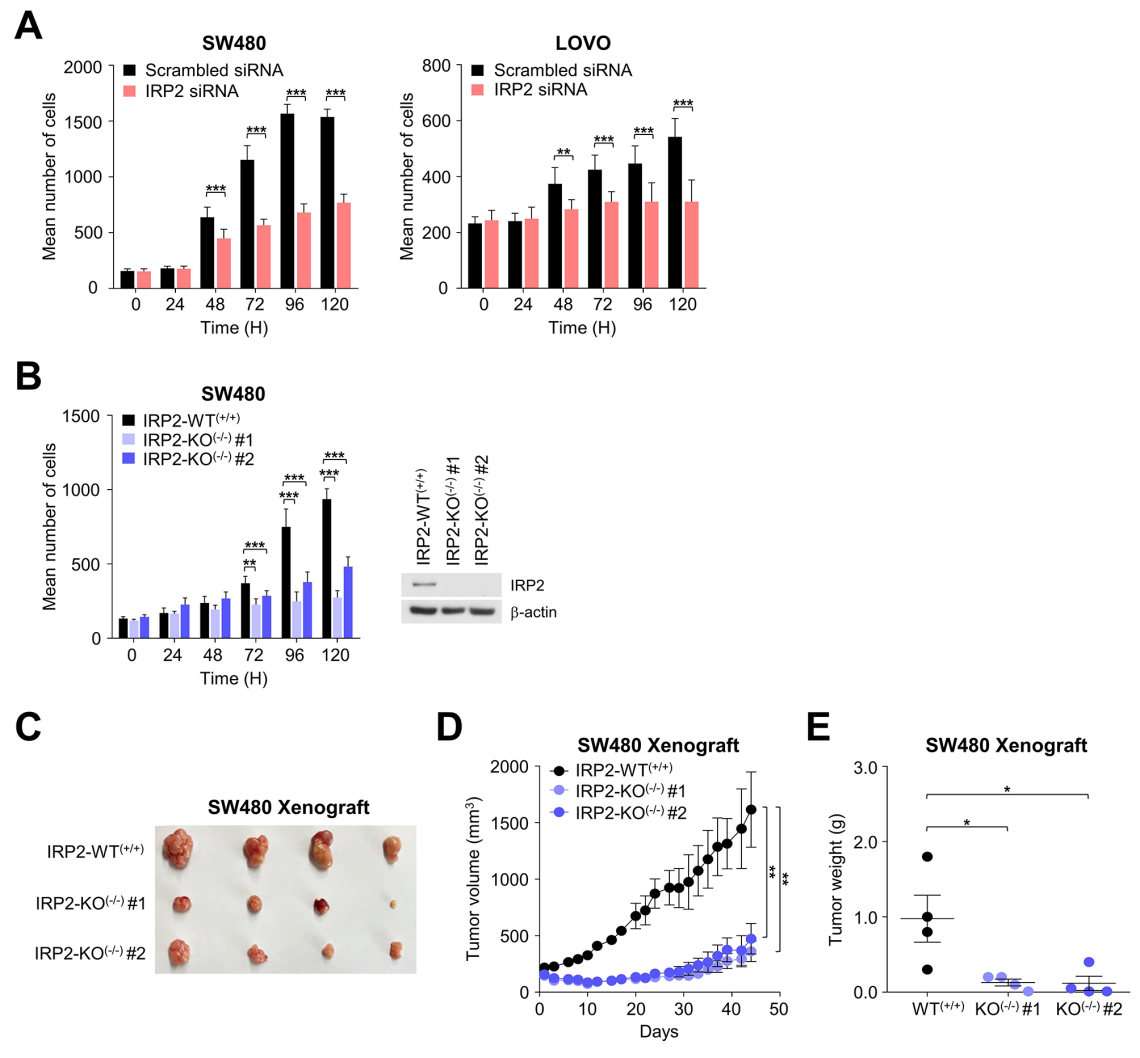
IRP2 regulated tumor cell growth in CRC cells. **(A)** Cell proliferation assay was performed on cells transfected with siRNA. Cells were monitored every 24 h, and six random fields were counted using the Harmony software. Data are represented as the mean ± SEM (*n* = 4). **p < 0.01, ***p < 0.001. **(B)** Cell proliferation assay was performed on IRP2 knockout cell lines. Cells were monitored every 24 h, and six random fields were counted using the Harmony software. IRP2 knockout was confirmed using immunoblotting. Data are represented as the mean ± SEM (*n* = 4). **p < 0.01, ***p < 0.001. **(C)** Representative tumor image of SW480 xenograft injecting IRP2 knockout cell (*n* = 4). **(D)** Tumor volumes were monitored once every two days for 44 days. Data are represented as the mean ± SEM (*n* = 4). **p < 0.01. **(E)** The tumors were isolated and weighed. Data are represented as the mean ± SEM (*n* = 4). *p < 0.05

Given the specific association of IRP2 with dysregulated iron metabolism in CRC, we explored its impact on cancer cell proliferation. Small interfering RNA (siRNA) and CRISPR-Cas9 were used to specifically target IRP2 for deletion. Our results revealed a significant reduction in the viability of IRP2-deficient cells, which decreased by more than 50% compared to control cells, substantiating IRP2’s role in promoting cancer cell survival (Fig. 1A and B). Furthermore, in vivo studies of IRP2 knockout (KO) in SW480 cells showed that the absence of IRP2 markedly delayed tumor formation compared to wild-type (WT) counterparts. The tumor growth inhibition (TGI) for IRP2 KO^(−/−)^ #1 cell was 77%, and for IRP2 KO^(−/−)^ #2 cell, it was 70% on day 44, with both results showing statistical significance (**p < 0.01) (Fig. 1C and D, and 1E). Overall, these results indicated that the suppression of IRP2 significantly delays tumor growth in CRC cells.

### High IRP2 expression linked to poor prognosis in CRC

**Fig. 2:**
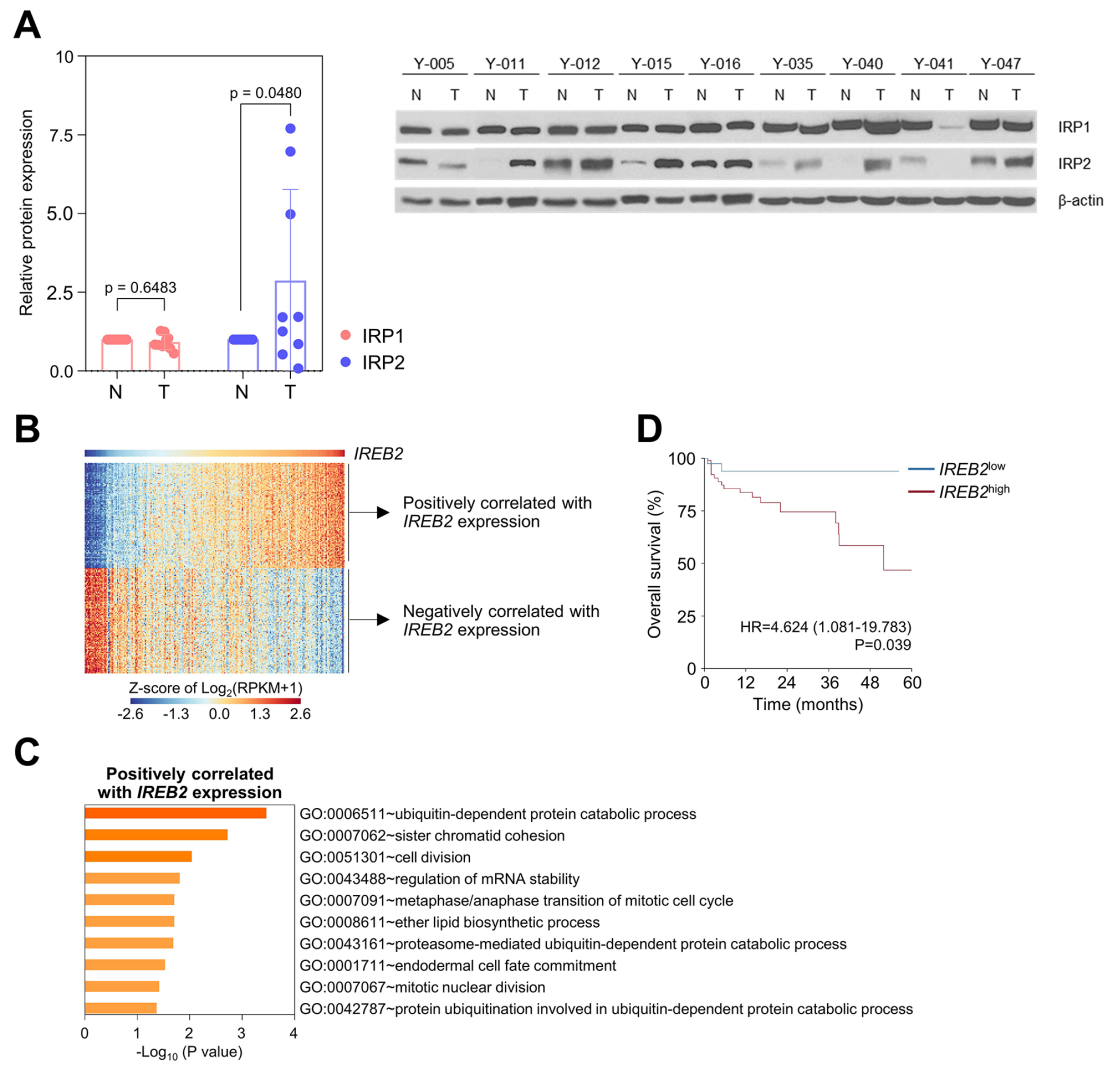
IRP2 is overexpressed and exhibited therapeutic relevance in colorectal cancer (CRC). **(A)** The expression level of IRP1 and IRP2 protein in normal (N) tissue (*n* = 9) versus colorectal tumor (T) tissues (*n* = 9) was measured using immunoblotting. Relative protein levels were calculated using the Image J software. **(B)** Heat map for top 100 genes which have positive or negative correlation with *IREB2* expression. CRC cases are shown according to *IREB2* expression level with the highest *IREB2* expression placed on the right side. The expression of selected genes is presented as row-wise z scores of log_2_ (RPKM + 1). **(C)** Gene Ontology analysis for the top 100 genes positively correlated with *IREB2*. **(D)** Kaplan–Meier curve comparing survival outcomes according to expression of *IREB2*. Patients were dichotomized based on cutoff value derived from log-rank maximization method

Cancer cells require comparatively more iron content than normal cells for division, proliferation, and metastasis [23]. To examine the effects of iron on cell growth, we initially used ferric ammonium citrate (FAC; formula (NH_4_)_5_[Fe(C_6_H_4_O_7_)_2_]) which triggers cellular iron abundance. We selected a 300 µM concentration of FAC to effectively examine the impact on tumor growth dynamics [7, 24, 25]. This resulted in increased growth of CRC cells (DLD-1, HCT116, HCT15, LOVO, SW480 and SW620) in a time-dependent manner; in contrast, treatment with deferoxamine (DFO), which induces iron depletion, displayed inhibitory effects on the growth of these CRC cell lines, as compared to the response seen in FAC-treated cells (Fig. S1). These results revealed that the cellular iron concentration is a critical factor determining the proliferation rate of cancer cells. We next evaluated the protein expression levels of IRP1 and IRP2 in paired samples of normal adjacent mucosa and tumor tissues from patients. After normalizing the paired normal tissue levels to a baseline of 1.0, the mean expression level of IRP1 protein in tumor tissues was 0.83 ± 0.132 (*p*-value = 0.6483) compared to normal tissues. In contrast, IRP2 protein exhibited a mean expression level of 0.87 ± 0.91 (*p*-value = 0.0480), as shown in Fig. 2A and Table S1. This substantial upregulation, with IRP2 levels approximately 3-fold higher in tumor tissues than in normal tissues, may reflect the role of IRP2 in tumorigenesis.

To explore the implication of IRP2 expression by analyzing The Cancer Genome Atlas (TCGA) CRC cohort [26]. When correlations between the expression of *IREB2* (gene name for IRP2) and various other genes were investigated, genes involved in ubiquitination (*ARIH1*, *BTBD1*, *BUB1B*, *CDC27*, *FEM1B*, *PSMA4*, *UBE2K*, *UBE3A*, *USP1*, *USP8*, and *USP45*) were ranked in the top 100 genes positively correlated with *IREB2* (Fig. 2B). Correspondingly, gene ontology term analysis indicated enrichment of ubiquitin-related pathways (GO0006511, GO0043161, and GO0042787) among those that were positively correlated with IRP2 expression (Fig. 2C). In addition, higher IRP2 expression was associated with worse overall survival (Fig. 2D), emphasizing the relevance of IRP2 as a therapeutic target in CRC.

### Identification of small molecules that inhibit the binding of IRP2 to IREs

**Fig. 3:**
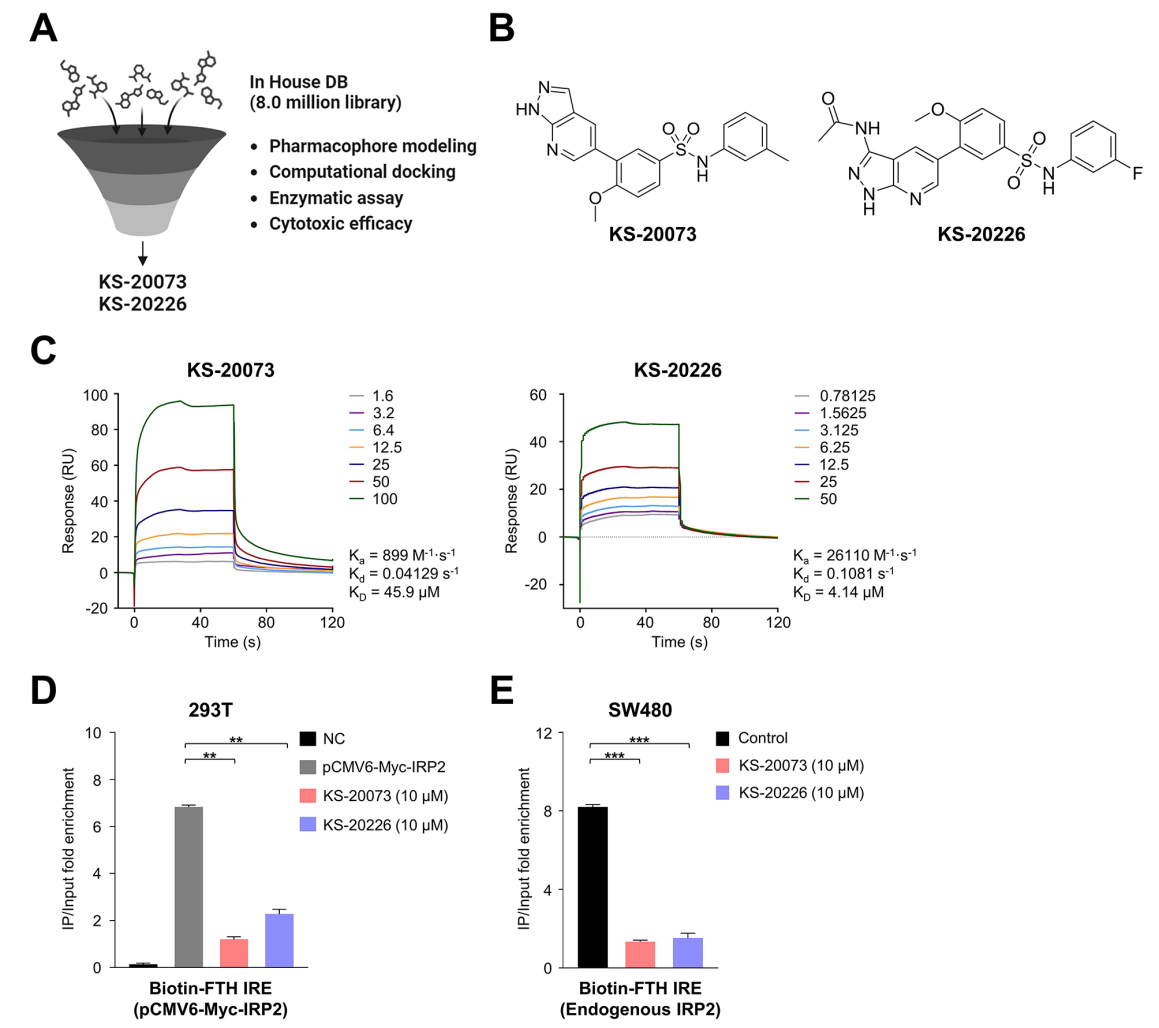
Identification of IRP2 inhibitors that dispersed the interaction between IRP2 and IREs. **(A)** Schematic diagram of the workflow implemented to identify small molecule inhibitors of IRP2. **(B)** Structures of KS-20073 and KS-20226. **(C)** Surface plasmon resonance (SPR) analysis revealed the inhibition of IRP2 binding to IRE by KS-20073 and KS-20226 in a dose-dependent manner. **(D)** Binding of IRP2 to biotin-FTH IRE was assessed using the RNA immunoprecipitation (IP) assay. HEK-293T cells were transfected with pCMV6-Myc (empty) and the pCMV6-Myc-IRP2 plasmid and were treated with KS-20073 and KS-20226 (10 µM) for 24 h. mRNA enrichment was achieved using qRT-PCR and normalized with input total RNA. Data are represented as the mean ± SEM (*n* = 4). **p < 0.01. **(E)** The RNA IP assay was used to assess the effect of endogenous IRP2 on IRP2 inhibitors. SW480 cells were treated with KS-20073 and KS-20226 (10 µM) for 24 h. Data are represented as the mean ± SEM (*n* = 4). ***p < 0.001

Building on this proof of concept, we identified small molecules that interfere with the binding of IRP2 to IREs (Fig. 3A). A virtual screening was performed using an in-house database (DB) containing 8.0 million compounds. In total, 5,782 compounds were screened during primary screening, and the subsequent filtering out on the based on the fitness score (above 1.0) and additional visual inspection resulted in a final selection of 32 compounds. Chemical modification led to the development of KS-20073 and KS-20226, which exhibit potent cytotoxic effects, with a growth inhibiting concentration 50 (GI_50_) value of 2.81 ± 0.275 µM and 3.18 ± 0.329 µM, respectively (Fig. 3B and S2A-2E).

Using SPR analysis, KS-20073 and KS-20226 were found to binding to IRP2, with K_D_ of 45.9 µM and 4.1 µM, respectively (Fig. 3C). These compounds effectively dispersed the interaction between IRP2 and IREs, which was substantiated by the RNA IP analysis (Fig. 3D and E). This led us to discover that IRP2 inhibitors substantially interfered with the IRP2–IRE complex through the structure modeling and an in vitro assay.

### IRP2 inhibitors induce cytotoxic effects through ubiquitin-dependent IRP2 degradation-mediated reprogramming of iron metabolism in CRC cells

**Fig. 4:**
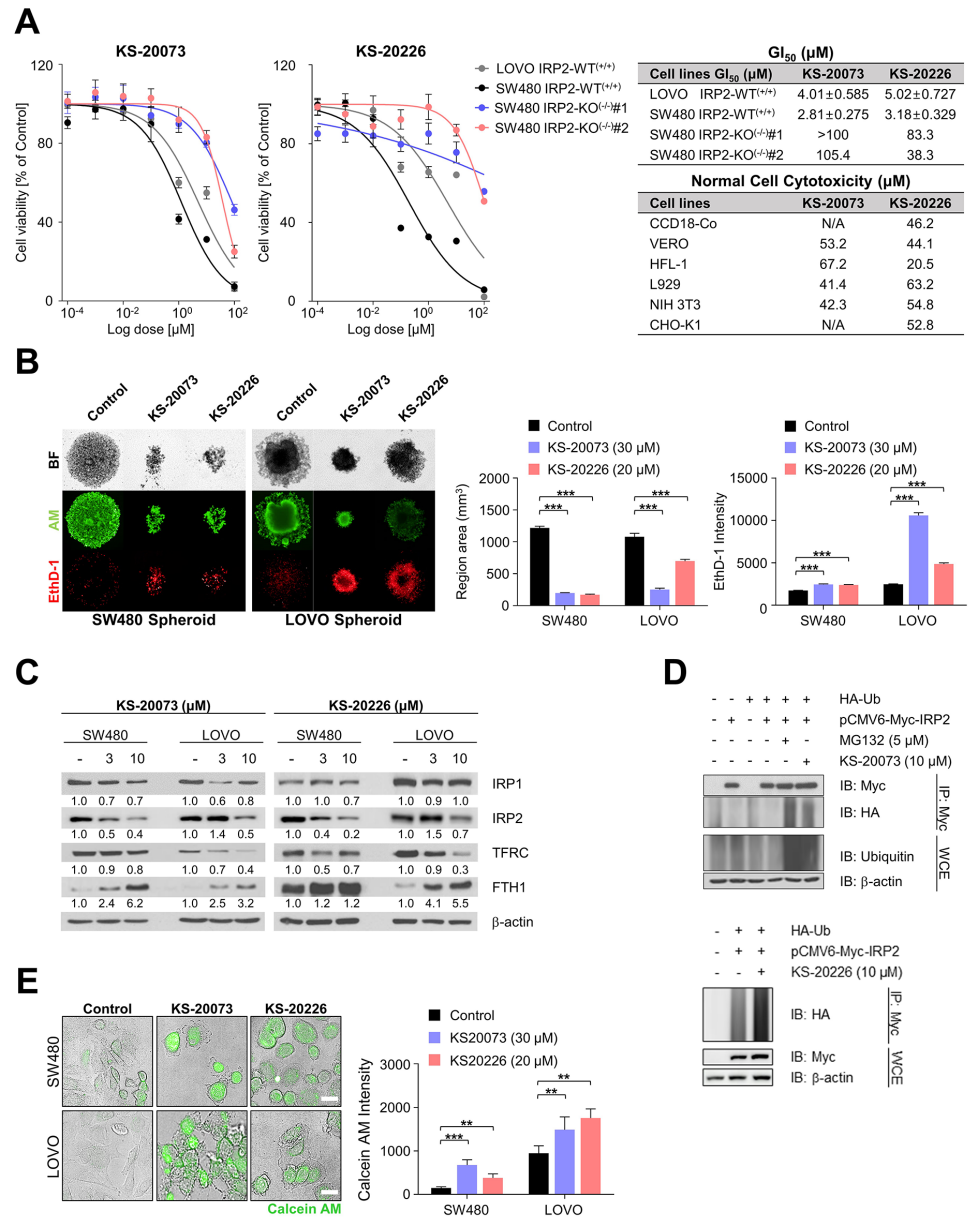
IRP2 inhibitors induce the ubiquitin dependent degradation of IRP2 which has anti-tumor activity. **(A)** Cytotoxicity of KS-20073 and KS-20226 in CRC cells and divergent normal cells. CCD18-Co; Human normal colon cell line, VERO; African green monkey kidney cell line, HFL-1; Human embryonic lung cell line, L929 (NCTC clone 929); Mouse fibroblast cell line, NIH 3T3; Mouse embryonic fibroblast cell line, CHO-K1; Chinese hamster ovary cell line. **(B)** 3D Spheroid toxicity of KS-20073 (30 µM) and KS-20226 (20 µM). For spheroid formation, cells were grown in U-bottom plates coated with poly-HEMA to maintain low adherent condition. Cells were treated with IRP2 inhibitors at days 3, 5, and 7 days, stained with Calcein AM (AM), and Ethidium homodimer-1 (EthD-1), and observed using confocal microscopy. The region area and fluorescence intensity of EthD-1 was assessed and quantified using the Harmony software. Data are represented as the mean ± SEM (*n* = 3). ***p < 0.001. **(C)** IRP1, IRP2, TFRC, and FTH1 protein expression levels were assessed via the immunoblotting of SW480 and LOVO cells treated with KS-20073 and KS-20226 (3 and 10 µM) for 72 h. β-actin was used as the protein-loading control. Relative protein levels were calculated using the Image J software. **(D)** HEK-293T cells were transfected with the HA-Ub and Myc-IRP2 plasmids for the ubiquitination assay. After 36 h of transfection, the cells were treated with KS-20073 (10 µM), KS-20226 (10 µM) and MG-132 (5 µM) for 24 h and subjected to immunoprecipitation. **(E)** Labile iron pool (LIP) was determined using Calcein AM (green). SW480 and LOVO cells were incubated with KS-20073 (30 µM) and KS-20226 (20 µM) for 24 h and then, quantified using the Harmony software. Data are represented as the mean ± SEM (*n* = 3). **p < 0.01, ***p < 0.001

Next, we evaluated the anti-proliferative activity of IRP2 inhibitors by measuring cell viability in CRC cells. These inhibitors exhibited remarkable cytotoxicity in an IRP2 dependent manner and had no effect on proliferation in the corresponding IRP2 knockout cells (Fig. 4A). By contrast, KS-20073 and KS-20226 showed minor cytotoxicity even at a high dose of 40 µM in different normal cell lines, such as CCD-18Co, VERO, VERO, HFL-1, L929, NIH 3T3, and CHO-K1 (Fig. 4A). Since three-dimensional (3D) culture models reflect the patient’s tumor tissue more accurately than 2D monolayer cell culture [27], we evaluated the growth inhibitory effect of KS-20073 and KS-20226 on SW480 and LOVO cells in a 3D spheroid model. Following 10 days of treatment, KS-20073 and KS-20226 displayed anti-proliferative effect, indicated by a reduction in the spheroid area and an increase in the number of dead cells stained with ethidium homodimer-1 (EthD-1; Fig. 4B). Moreover, cell cycle analysis revealed that KS-20073 and KS-20226 induced a significant accumulation of cells in the G2/M phase, which was accompanied by a decrease in cell accumulation in the G0/G1 phase (Fig. S3A), suggesting the occurrence of G2/M arrest.

We examined whether KS-20073 and KS-20226 disrupted iron metabolism through the reduction of IRP2, considering that it targets IRP2 binding to IREs. These IRP2 inhibitors were found to eliminate IRP2 expression at the protein level, resulting in decreased iron uptake with enhanced iron storage capacity by modulating TFRC and FTH1 expression (Fig. 4C). These inhibitors did not alter IRP1/2 transcription (Fig. S3B), suggesting that KS-20073 and KS-20226 modulate IRP2 expression at the translational or post-translational level rather than the transcriptional level.

To investigate the mechanisms of iron metabolism disruption following IRP2 degradation, we performed ubiquitination analysis. KS-20073 and KS-20226 enhanced the ubiquitination of IRP2 to a similar extent as MG-132, a proteasome inhibitor triggering unselective ubiquitination (Fig. 4D). Since the KS-20073 and KS-20226-induced TFRC downregulation affects intracellular iron absorption, we expected that the labile iron pool (LIP) decreases upon the degradation of IRP2 protein by IRP2 inhibitors. The fluorescence of Calcein acetoxymethyl ester (AM), a LIP determination probe, is quenched following chelation of labile iron, and the degree of quenching gives an estimated amount of chelatable iron. The intensity of Calcein AM increased following treatment with KS-20073 and KS-20226, di-2-pyridylketone-4-cyclohexyl-4-methyl-3-thiosemicarbazone (DpC), and DFO, an iron chelator that diminishes free iron through forming complex interactions with iron (Fig. 4E and Fig. S4). Collectively, these results indicated that the ubiquitin-dependent degradation of IRP2 disrupted the maintenance of iron homeostasis following LIP reduction.

### Inhibition of IRP2 leads to mitochondrial dysfunction and autophagy via the AMPK-ULK1-Beclin1-LC3B cascades

**Fig. 5:**
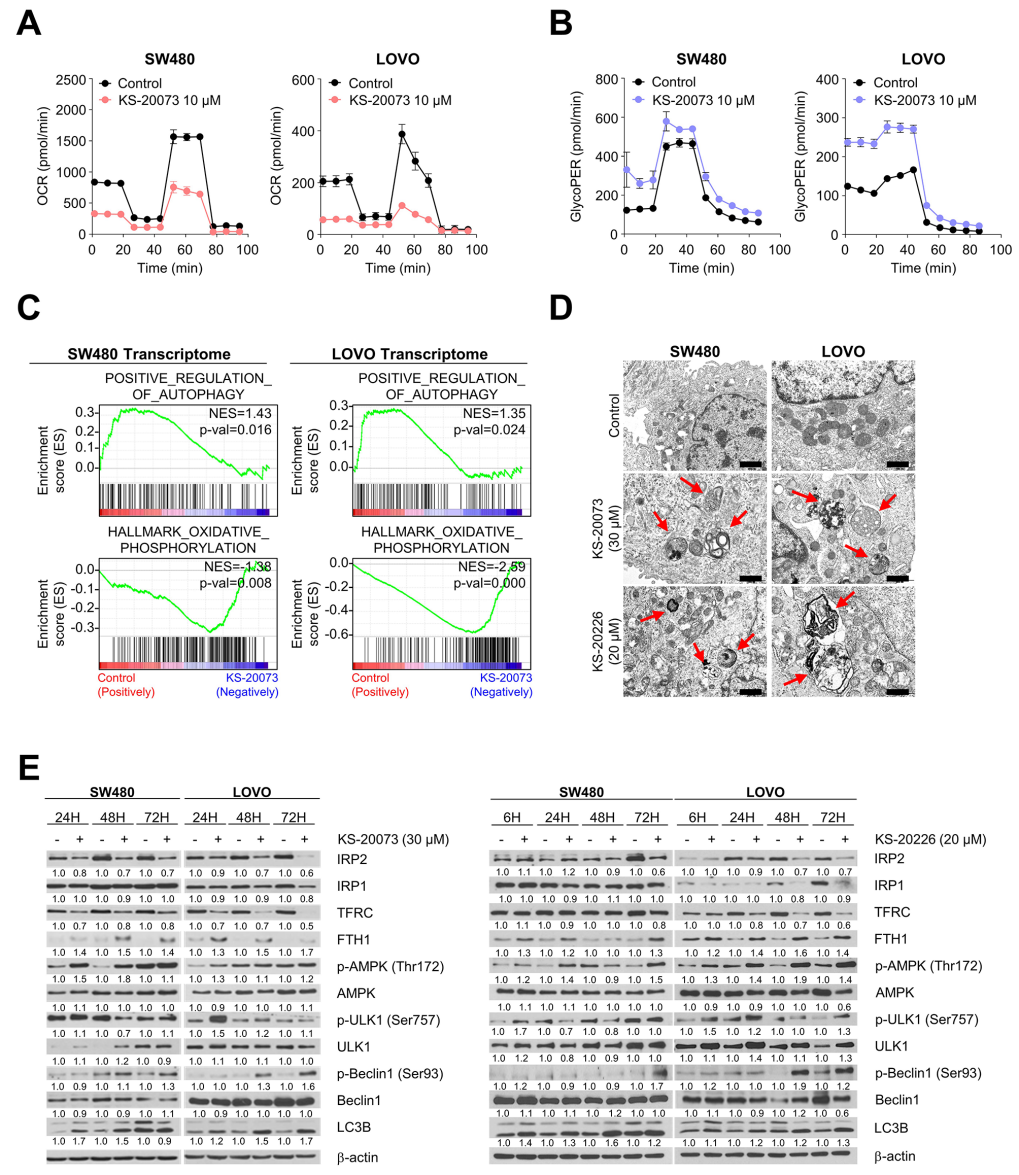
Silencing IRP2 leads to sequential reprogramming of mitochondrial metabolism and autophagy. **(A)** Effects of KS-20073 (10 µM) on oxygen consumption rate (OCR). Data are represented as the mean ± SEM (*n* = 3). **(B)** Effects of KS-20073 (10 µM) on glycolysis. Data are represented as the mean ± SEM (*n* = 3). **(C)** GSEA plots with selected genes are shown. GSEA showing an overrepresentation of the “POSITIVE REGULATION OF AUTOPHAGY”, underrepresentation of the “OXIDATIVE PHOSPHORYLATION”. **(D)** Representative transmission electron microscopy (TEM) images of IRP2 inhibitors (KS-20073; 30 µM and KS-20226; 20 µM) for 48 h. The red arrowheads indicate the autophagosomes. Scale bars in figures are 2000 nm. **(E)** Time-dependent expression of autophagy related proteins by KS-20073 (30 µM) and KS-20226 (20 µM). Relative protein levels were calculated using the Image J software

Since the IRP2 inhibitors caused an iron deficiency via perturbation of iron metabolism, we predicted that it would also affect mitochondrial function. KS-20073 undermined mitochondrial oxidative phosphorylation (OXPHOS), resulting in the repression of oxygen consumption rate (OCR), followed by a reduction in basal respiration and ATP production (Fig. 5A and S5A). Conversely, KS-20073 activated glycolysis, indicating an increase in both basal and compensatory glycolysis (Fig. 5B and S5B).

To investigate the global effects of the small molecule IRP2 inhibitors in CRC cells, we further performed Gene Set Enrichment Analysis (GSEA) from RNA sequencing data. KS-20073 considerably suppressed the genes involved in oxidative phosphorylation and enriched the expression of genes involved in regulation of autophagy, suggesting a shift in cellular energy metabolism towards a state that favors autophagic processes over mitochondrial respiration (Fig. 5C). Initially identified as an IRP2 inhibitor, KS-20073 formed the basis of our mechanistic studies, but subsequent development led to the discovery of KS-20226, a chemically analogous compound with enhanced efficacy, which we incorporated into later experiments.

Dysfunctional mitochondria undergo mitophagy which is the selective degradation of mitochondria by autophagy [28]. Mitochondrial dysfunction stimulates AMP-activated protein kinase (AMPK), which leads to the activation of Unc-51-like autophagy activating kinase-1 (ULK1) and Beclin-1, which act as accelerators of autophagy [29]. This suggests that disturbance of iron metabolism by targeting IRP2 leads to activation of autophagy through dysfunctional mitochondria.

Having observed the transcriptional activation of autophagy target genes by IRP2 inhibitors, we next asked whether the inhibition of IRP2 practically triggered autophagy. Genetic ablation of IRP2 in SW480 cells resulted in the formation of cellular multi-vesicles and autophagosomes, leading to autophagic cell death similar to that induced by pharmacologic inhibition using KS-20073 and KS-20226, as observed using transmission electron microscopy (TEM) (Fig. 5D and S5C). Additionally, pharmacologic suppression of IRP2 by KS-20073 and KS-20226 activated AMPK phosphorylation, and the expression of ULK1 and Beclin-1 sequentially elevated after treatment with the IRP2 inhibitors in a time-dependent manner (Fig. 5E). Finally, we assessed the expression of LC3B, a central marker of autophagy, using immunostaining. As shown in Fig. S5D, KS-20073 and KS-20226 remarkably increased LC3B expression, suggesting that IRP2 inhibition leads to CRC cell death relying on activation of the AMPK-ULK1-Beclin1 pathway.

### Suppression of IRP2 shows divergent sensitivity in CRC organoids and suppresses tumor growth in vivo

**Fig. 6:**
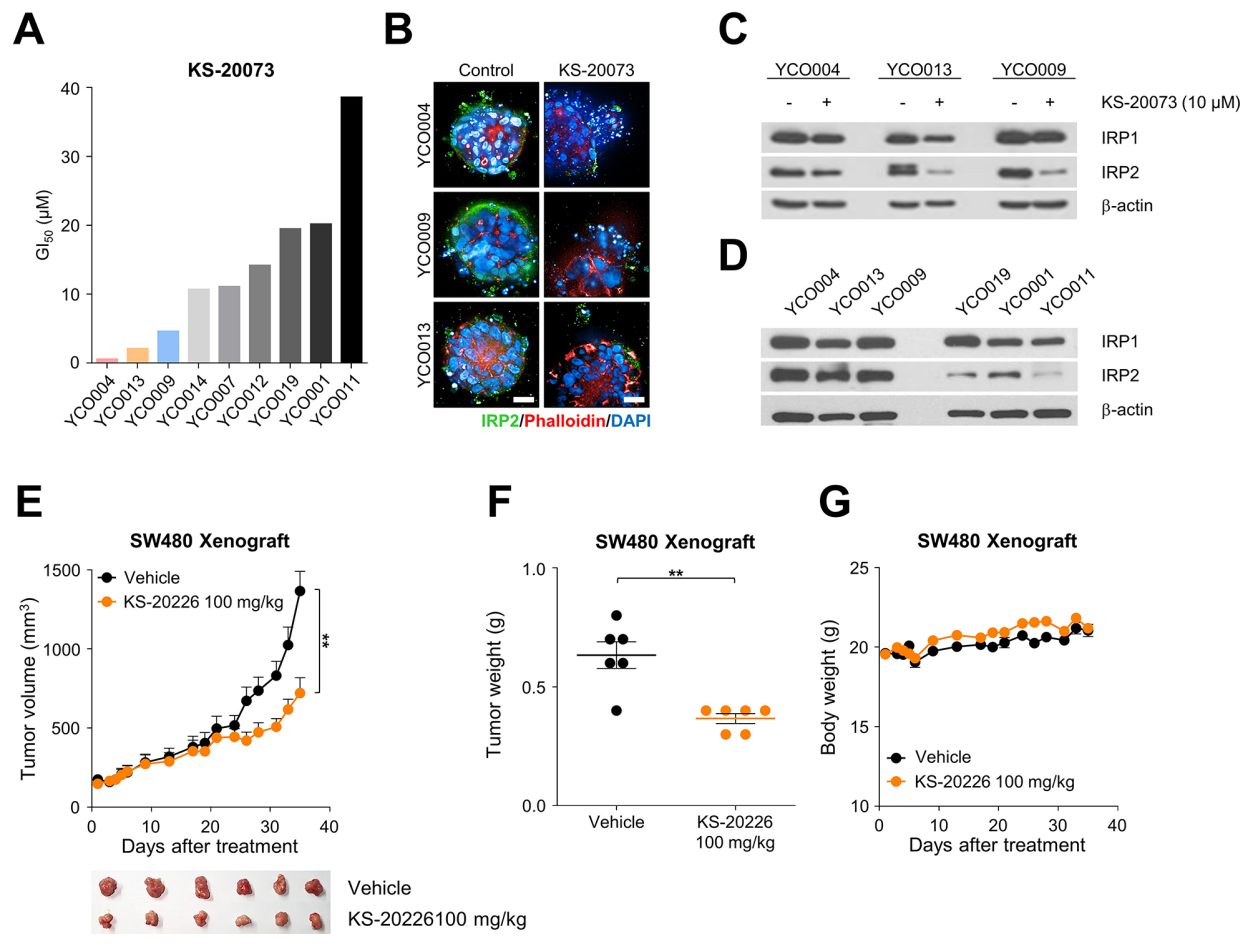
Suppression of IRP2 delayed tumor growth in CRC organoids and in vivo model. **(A)** Cytotoxic effect of KS-20073 on CRC organoids. Organoids were treated with KS-20073 for 5 days and subjected to the cytotoxic analysis using CellTiter-Glo. **(B)** Representative immunofluorescence images of IRP2 (green), Phalloidin (red) and DAPI (blue) in KS-20073-sensitive organoids by treatment of KS-20073 (10 µM) were obtained using confocal microscopy. **(C)** IRP1, IRP2 and β-actin protein levels in KS-20073-sensitive organoids were measured using immunoblotting. **(D)** The protein levels of IRP1 and IRP2 were determined via immunoblotting using organoids most sensitive to KS-20073 vs. the less sensitive organoids to KS-20073. **(E)** Tumor volumes and images of SW480 xenografts injected intraperitoneally with vehicle or KS-20226 (100 mg/kg). Data are represented as the mean ± SEM (*n* = 6). **p < 0.01. **(F)** Tumor weights of SW480 xenografts treated with vehicle or KS-20226 (100 mg/kg). Data are represented as the mean ± SEM (*n* = 6). **p < 0.01. **(G)** Body weight after treatment with KS-20226 (100 mg/kg) for 36 days

We treated nine established CRC organoids with different concentrations of KS-20073, and monitored the changes in morphology as well as measured cell viability using CellTiter-Glo. KS-20073-treated organoids exhibited the disappearance of the crypt as well as the disruption of intact organoid structure, with differential susceptibility ranging from an GI_50_ of 0.5 to 40 µM (Fig. 6A). We classified the organoids into those with the highest and lowest sensitivities for KS-20073 for further study. Furthermore, KS-20073 markedly eliminated IRP2 protein expression in the organoids most sensitive to the inhibitor (Fig. 6B and C). Notably, IRP2 expression levels correlated with susceptibility of the organoids to KS-20073 (Fig. 6D).

Finally, we assessed the anti-tumor activity of KS-20073 and KS-20226 in vivo using xenograft mouse models established by subcutaneously injecting the SW480 cell line. Tumor volumes were significantly reduced after intraperitoneal administration of KS-20073 and KS-20226 (100 mg/kg) (Fig. 6E and S6A). The administration of the IRP2 inhibitors effectively suppressed tumor weight (Fig. 6F and S6B) and had no significant impact on body weight (Fig. 6G and S6C). To further verify the results of the in vitro assay, tumors were subjected to immunoblotting. Collectively, these results suggested that the novel small molecule inhibitors targeting IRP2 showed effective tumor inhibitory responses in vivo.

## Discussion

Targeting iron metabolism, which is involved in tumor initiation and progression, is an attractive strategy for cancer treatment [30, 31]. However, efforts to utilize nonselective iron chelating agents to disrupt systemic iron metabolism are accompanied with inevitable toxicity and have been largely unsuccessful [32]. IRPs are master regulators of intracellular iron homeostasis, which is involved in various biological processes [33]. The IRP2 inhibitors we developed regulate iron metabolism, interfering with the IRP2-mediated occupancy of IREs and induced ubiquitin-mediated IRP2 degradation. Inhibition of cancer growth was achieved via cell death driven by IRP2 inhibitors, while minimizing the impact on systemic iron metabolism. Furthermore, IRP2 expression was found to be correlated with the efficacy of IRP2 inhibitors, providing plausible predictive markers for these compounds.

Dependency on iron metabolism in promoting cancer stemness and growth has been exploited for the development of iron-depleting agents as therapeutic strategies for cancer treatment. For instance, water-soluble iron chelators, such as DFO and deferasirox [34], and lipid-soluble iron chelating agents, such as Dp44mt and DpC [35], have shown marked efficacy in vitro. However, usage of these compounds involves significant drawbacks, including poor penetration through the cell membrane and generation of ROS by chelating not only divalent metal ions but also copper, iron, and manganese [36], all of which are associated with significant toxicity with reduced efficacy. Additionally, the nonselective nature of these compounds perturbs systemic iron metabolism, limiting their clinical usefulness. In this study, we discovered that IRP2 selectively regulated iron metabolism in CRC with prognostic implications. Unlike IRP2, IRP1 interconverts between two distinct forms: a cytosolic aconitase and an IRE-binding apo form. This unique characteristic means that the protein levels of IRP1 do not directly reflect its activity as an IRE-binding protein, which is a critical factor in iron metabolism regulation.

Building on this proof of concept, we initially designed the screening protocol to identify compounds that inhibit the binding of IRP2 to IREs. Subsequently, we hypothesized that inhibition of this binding disrupts IRP2 stabilization, as the protein is typically regulated through feedback mechanisms involving its interaction with IRE elements. When this interaction is obstructed, IRP2 may be tagged more frequently for degradation via the ubiquitin-proteasome pathway. Furthermore, it is crucial to understand that the GI_50_ values for IRP2 inhibitors represent the concentration required to achieve a 50% reduction in activity, which does not imply a complete displacement of binding interactions between the inhibitor and its target. In addition, in Figs. 4 and 5, varied drug concentrations were used to demonstrate the efficacy of the compounds across different conditions. Lower concentrations allowed us to observe cellular effects and iron metabolism changes with minimal cytotoxicity, while higher concentrations helped investigate the mechanistic impact on IRP2 ubiquitination and related processes, providing a comprehensive evaluation of the biological actions of the compounds.

The dissociation constant (Kd) values for IRP2 inhibitors in binding to IRP2 are indeed significantly higher than the affinity typically observed between IRP1, IRP2 and IREs, suggesting these small molecules have a much lower affinity for IRP2 compared to the natural interaction between IRP2 and IREs. However, there are several factors that could enable these small molecules to effectively outcompete the endogenous IREs despite their lower affinity. The effectiveness of small molecules can be influenced by their concentration relative to that of IREs. If these compounds are used at substantially higher concentrations, they may still be able to outcompete IREs for IRP2 binding despite their lower affinity. Furthermore, these compounds might have better cellular uptake or distribution within the cell, allowing them to localize efficiently to the sites where IRP2 is present. This spatial advantage could help them outcompete IREs despite their lower intrinsic affinity.

The pharmacologic inhibition of IRP2 exerted anti-tumor efficacy in various models, including patient-derived cell lines, organoids, and in vivo systems. Mitochondria are the central organelle for iron utilization to maintain numerous biological functions [37]. Fe-protoporphyrin (known as heme) and Fe-S clusters are two essential components of the inner membrane complexes of the mitochondrial electron transport chain [38]. Dysregulated iron metabolism causes diverse mitochondrial diseases affecting red blood cells [39], cardiomyocytes [40], and neuronal cells [41]. In cancer cells, mitochondrial iron metabolism is highly upregulated owing to their proliferative nature [42], resulting in a high demand for iron. In our study, pharmacologic inhibition of IRP2 reduced OXPHOS, suggesting that mitochondrial metabolism can be reshaped via IRP2 inhibition. Disruption of mitochondrial metabolism by IRP2 inhibitors was linked to autophagy via the AMPK-ULK1-Beclin1 pathway [43], highlighting the unique properties of IRP2 inhibition.

Our study has highlighted several critical limitations associated with the IRP2 inhibitors, indicating essential areas for enhancement. Firstly, the differentiation in the effects of the inhibitors on IRP1 versus IRP2 was unclear, highlighting the need for more precise experiments to isolate their distinct mechanisms. Additionally, the binding assays suggested that our inhibitors might not only prevent IRP2 binding to IREs but could also affect protein expression, requiring verification through advanced molecular docking and interaction studies. The notably low binding affinities of our compounds raise concerns about their ability to effectively compete with natural IREs. KS-20073 and KS-20226 effectively inhibited IRP2 expression and tumor cell growth in vitro, but showed limited efficacy in animal models at high concentrations due to poor pharmacokinetics and low solubility. To address these issues, further optimization of their pharmacokinetic profiles and solubility is essential. Future research should focus on developing improved analogs with better PK properties and higher solubility to enhance bioavailability and therapeutic efficacy in vivo. Optimizing these compounds will improve their therapeutic potential and contribute to more effective cancer treatments.

## Materials and methods

### Cell culture

The human CRC cell lines were obtained from Korean Cell Line Bank (Korea). Cell lines were maintained in specific complete growth media. Particularly, Dulbecco’s modified Eagle’s Medium (Lonza, SH30243.01) and Roswell Park Memorial Institute-1640 (Lonza, SH30027.01), containing 10% fetal bovine serum, 100 U/mL penicillin, and 100 µg/mL streptomycin, were used for the maintenance of cell lines. Cell lines were incubated at 37 °C in a 5% CO_2_ incubator.

### 3D spheroid culture

For spheroid formation, 100 µL/well of cell suspensions at optimized densities (SW480; 1 × 10^2^ and LOVO; 1 × 10^3^) were dispensed into U-bottom 96-well plates coated with 2-hydroxyethyl methacrylate (poly-HEMA) (Sigma, P3932) for low attachment. Plates were incubated for three days at 37 °C, and spheroids were treated with KS-20073 and KS-20226 thrice every two days. Next, spheroids were stained with Hoechst33342 (Invitrogen, H1399) to observe nuclei, Calcein AM (Invitrogen, C3099) to observe live cells, and ethidium homodimer (Invitrogen, E1169) to identify dead cells and images were taken using Operetta CLS (PerkinElmer).

### Organoid culture and viability

Approximately 1 cm^2^ of human colorectal tumor tissue was cut into small fragments and washed in phosphate-buffered saline (PBS) (Tech and Innovation, BPB-9111). Tissues were subjected to digestion by medium containing 1% fetal bovine serum and 625 µg/mL collagenase (Sigma, C9407) for 30 min at 37 °C. Next, tissues were centrifuged at 3,000 ×g for 3 min. Supernatants were discarded and washed with PBS. Cells were again centrifuged at 3,000 ×g for 3 min and seeded into Matrigel (Corning, 354230). The human CRC organoid medium contained the following essential components in Dulbecco’s modified Eagle’s medium/F-12 (Lonza, 12–719 F); 100 units Penicillin/Streptomycin (Gibco, 15140-122), 1× Glutamax (Gibco, 35050-061), 1× N2 supplement (Gibco, 17502-048), 1× B27 supplement (Gibco, 17504-044), 1 mM N-acetyl-L-cysteine (Sigma, A7250), 2 mM L-glutamine (Sigma, G7513), 10 mM nicotinamide (Sigma, N0636), 10 nM gastrin I (Sigma, G9145), 10 µM SB202190 (Sigma, S7067), 500 nM A83-01 (Sigma, SML0788), 50 ng/mL human epidermal growth factor (Peprotech, 100−47), 100 ng/mL human noggin (Peprotech, 120–10C). Organoids were passaged every 1–2 weeks at a 1:4 or 1:5 ratio via mechanical dissociation. For the cancer organoid viability assay, organoids were seeded with 10 µL Matrigel matrix dome into 96-well plates. The matrix domes were allowed to solidify for 30 min at 37 °C and treated with various concentrations of KS-20073 for 5 days. The CellTiter-Glo 3D cell viability reagent (Promega, G9683) was then added to the organoids followed by incubation for 30 min at 37 °C. The luminescence was measured using a microplate reader (Molecular Devices, FlexStation 3). Experiments were performed in triplicate, and the IC_50_ values calculated using the GraphPad Prism (5.0) software (La Jolla, CA, USA).

### Xenograft experiments

Male BALB/c nude mice, 5-week-old, were purchased from ORIENTBIO Inc. and housed under specific pathogen-free conditions with standard rodent chow and water and 12-h light-dark cycle. All experiments were conducted according to the institutional ethical guidelines on animal care and were approved by the Department of Laboratory Animal Resources, Yonsei Biomedical Research Institute. Cells were implanted into mice via subcutaneous injection into the right flanks at a concentration of 1 × 10^7^ cells/mouse (10 mice per group). Twenty mice with a tumor volume of 100–150 mm^3^ with no abnormalities in general health were selected and randomly allotted to a vehicle, KS-20073 100 mg/kg, or KS-20226 100 mg/kg group. Treatments were prepared in a mixture of 10% dimethyl sulfoxide (DMSO) (Sigma, D8418), 30% polyethylene glycol 400 (Sigma, 8.07485), and 60% PBS. Experiments were conducted daily using intraperitoneal injection of 100 mg/kg. During the treatment period, tumor volume was measured thrice per week according to the following formula: Tumor volume (mm^3^) = L (mm) × W^2^ (mm^2^) × 1/2. Body weights were monitored daily during the treatment period, and the final tumor weights were measured after sacrificing the mice on day 30 owing to planned termination of the experiment.

### SPR analysis

IRP2 (Theoretical pI: 6.5; Molecular weight: 97 kDa) was dissolved in 10 mM sodium acetate (pH 4.5) at a concentration of 50 µg/mL and injected onto the chemically activated surface of a CM5 sensor chip until the response units reached between 8,000 and 9,000 in running buffer (PBS-P [10.1 mM Na_2_PO_4_, 1.8 mM KH_2_PO_4_, 137 mM NaCl, 2.7 mM KCl, pH 7.4, 0.005% P20] supplemented with 2% DMSO). In SPR multi-cycle kinetic experiments, a twofold concentration series of KS-20073 or other KS compounds ranging from 1.6 to 100.0 µM were injected over the sensor surface at a rate of 30 µL/min, with 60 s contact at 25 °C and dissociation times without a regeneration step. A blank injection was used to minimize the carryover effects. The association (k_a_), dissociation (k_d_), and equilibrium dissociation (K_D_; k_d_/k_a_) constants were calculated from the sensorgrams by fitting with a 1 : 1 binding model using the evaluation software (v2.0) provided with the Biacore T200 instrument.

### siRNA transfection

siRNA transfection was performed using Lipofectamine 3000 reagent (Invitrogen, L3000) following the manufacturer’s instruction. Briefly, an appropriate number of cells (2 × 10^5^) were seeded in 6-well plates and incubated for 24 h at 37 °C. For transfection of each well, 5 µL of Lipofectamine 3000 was mixed with 125 µL of Opti-MEM. In a separate tube, the negative control and siIRP2 were added to 125 µL of Opti-MEM and the siRNA solution was added to the Lipofectamine 3000 mixture. The siRNA mixture was incubated for 15 min at room temperature to allow complex formation. Subsequently, the solution was added to the cells and incubated at 37 °C.

### CRISPR/Cas9 and transfection

The following insert oligonucleotides for human IRP2 guide RNA (gRNA) were used:

IRP2 gRNA #1 forward primer: 5′-CACCGGATTAATCTGAATTCAATAG-3′;

IRP2 gRNA #1 reverse primer: 5′- AAACCTATTGAATTCAGATTAATCC-3′;

IRP2 gRNA #2 forward primer: 5′- ACCGCAGGATAGAGTTGCTGTGAC-3′;

and IRP2 gRNA #2 reverse primer: 5′- AAACGTCACAGCAACTCTATCCTGC-3′.

The complementary oligonucleotides for guide RNAs (gRNAs) were ligated and cloned into the PX459 CRISPR/Cas9-Puro vector (Addgene, 48139). The SW480 and LOVO cells were transfected with either PX459/gRNA#1 or PX459/gRNA#2 using Lipofectamine 3000 according to the manufacturer’s protocol. Two days after transfection, the cells were treated with 1 µg/mL puromycin (Sigma, P8833) thrice per week. After two weeks, colonies were isolated and analyzed using immunoblotting.

### Cell viability and proliferation assay

Cell viability was determined using the Cell Counting Kit-8 (Dojindo, CK04). An appropriate number of cells (2 × 10^4^) were seeded in 96-well plates and incubated for 24 h at 37 °C. Cells were then treated with various concentrations of the compounds for 48 h. Next, the CCK-8 solution was added to the cells, followed by incubation for 3 h at 37 °C. The absorbance was measured at 450 nm using a microplate reader (Molecular Devices, Versamax). Experiments were performed in triplicate.

For the proliferation assay, cells (1 × 10^5^) were seeded in a 6-well plate and incubated at 37 °C for 24 h. The medium in each well was then replaced with KS-20073 and KS-20226 in fresh culture media. Cells were monitored every 12–24 h for 5 days and counted using Operetta CLS. Experiments were performed in triplicate.

### RNA immunoprecipitation assay

HEK293T cell was transfected with the pCMV6-Myc-IRP2 plasmid (Origene, PS100016) for 36 h and then, treated with KS-20,073 for 24 h. After incubation, the medium was removed, followed by cross-linking using formalin (Tech and Innovation, BPP-9004). Cells were lysed in RNA immunoprecipitation lysis buffer (25 mM Tris-HCl [pH 7.4], 150 mM KCl, 5 mM EDTA, 0.5% NP-40 [Sigma, I8896], 0.5 mM DTT [Sigma, 10197777001], 100 U/mL RNase inhibitor [Invitrogen, AM2696] and Complete Mini Protease inhibitor cocktail [GeneDepot, P3100]). Lysates were aliquoted to obtain input samples. Then, cell lysates were mixed with Dynabeads Protein G (Invitrogen, 10003D) pre-incubated with anti-Myc antibody (Santa Cruz Biotechnology, sc-40) and incubated at 4 °C overnight with gentle rotation. The beads were immobilized, washed five times with NT-2 buffer (50 mM Tris-HCl [pH 7.4], 150 mM NaCl, 1 mM MgCl_2_, 0.5% NP-40, and Complete Mini Protease inhibitor cocktail), and mixed with Trizol (Ambion, 15596018) for RNA purification according to the manufacturer’s instructions, followed by quantitative reverse transcription-PCR (qRT-PCR) using FTH or 18 S primers. RNA enrichment was normalized with input total RNA, and experiments were performed in triplicate.

### Ubiquitination assay

For the ubiquitination assay of IRP2, HEK293T cells grown on 10-cm dish were co-transfected with 10 µg of myc-tagged IRP2 and a vector expressing HA-tagged ubiquitin (Addgene, 18712) each for 48 h. Following transfection, MG-132 (5 µM) (Sigma, M8699) was added to the cells for 24 h to inhibit proteasomal degradation, followed by KS-20073 and KS-20226 (10 µM). Cells were harvested in lysis buffer containing 0.5% NP-40. Immunoprecipitation was performed in PBS using 10 µg of an anti-Myc antibody and 300 µg of the cell lysate. Immunoprecipitates were resolved using 8% sodium dodecyl sulfate polyacrylamide gel electrophoresis (SDS-PAGE) and probed with an anti-HA antibody (Santa Cruz Biotechnology, sc-7392) to monitor IRP2 ubiquitination.

### qRT-PCR

Total cellular RNA was isolated used to synthesize cDNA. qRT-PCR was conducted on an Applied biosystems 7500 apparatus using SYBR green master mix (Applied biosystem, 4368708) according to the manufacturer’s instructions. The housekeeping gene β-actin was used as the endogenous control for normalization. The following qRT-PCR primers were used:

IRP1 forward primer: 5′-CGCTGTGGTTGACTTTGCTGCAATG-3′;

IRP1 reverse primer: 5′-ATCTATTACAAGATCAGCAGGGCAG-3′;

IRP2 forward primer: 5′-GGCTGCAGAGCTGTACCAGAAAGAA-3′;

and IRP2 reverse primer: 5′-CGGTCCTTTGGCAGCCCAGTCTCTG-3′.

### Immunoblotting and immunofluorescence staining

For immunoblotting, cells were lysed using radio immunoprecipitation lysis buffer, containing a protease inhibitor and a phosphatase inhibitor cocktail (GenDepot, P3200), to extract proteins. After centrifugation at 13,000 rpm for 20 min at 4 °C, the supernatants were collected and protein concentrations were determined using Bradford assay. Approximately, 30–50 µg proteins were size-fractionated using 8–15% SDS-PAGE and transferred onto a polyvinylidene difluoride membrane by semi-transfer. Membranes were blocked with 5% non-fat milk in PBS with 0.1% tween-20 for 1 h at room temperature and incubated with primary antibodies overnight at 4 °C. The following antibodies were used for immunoblotting; IRP1 (Santa Cruz, sc-166022, 1:500) and IRP2 (Santa Cruz, sc-33682, 1:500). After washing with PBS, the membranes were incubated with horseradish peroxidase-conjugated secondary antibodies for 1 h at room temperature. Immuno-reactive bands were visualized using an enhanced chemiluminescence reagent (ELPIS Biotech, EBP1071).

For immunofluorescence staining, cells were grown under cell culture conditions on 96-well plates for 24 h and treated with KS-20073 for 72 h. Next, cells were washed with PBS and fixed in cold-methanol for 5 min. After fixation, the cells were incubated with a mouse monoclonal antibody against IRP2 overnight at 4 °C. Cells were incubated with the Alexa Fluor 488 goat anti-mouse secondary antibody (Invitrogen, A11001) for 2 h and nuclei were counterstained with DAPI (Sigma, D9542) for 5 min. Cells were washed thrice with PBS and observed using Operetta CLS.

### Cell cycle analysis

To analyze cell cycle distribution in response to the KS-20073 and KS-20226 treatment, SW480 and LOVO cells were treated with KS-20073 and KS-20226 for 48 h. Cells were harvested using trypsinization, washed with PBS, and re-suspended thoroughly in 300 µL PBS. The cells then fixed in 700 µL of 100% ice-cold ethanol for 2 h at − 20 °C. Fixed cells were mixed with PI/RNase Staining Solution (BD Biosciences, 550825) for 30 min. Cell cycle analysis was performed on BD FACS LSR II system.

### LIP determination

LIP assay was measured using Calcein-AM. The cells were loaded with 2 µM Calcein AM for 30 min at 37 °C and then washed with PBS. Fluorescence was measured at 485 nm excitation and 535 nm emission wavelengths using Operetta CLS.

### Measurement of OCR and extracellular acidification rate

SW480 and LOVO cells were seeded and treated with KS-20073 for 24 h. Cells were seeded at a density of 4 × 10^4^ cells/well in a XF^e^24 microplate coated with 0.2% gelatin and determined using the XF cell energy phenotype test. The cells were washed twice, and medium was replaced with the XF assay medium containing 4.5 g/L glucose, 4.0 mM glutamine, and 1.0 mM sodium pyruvate. The plates were placed in a 37 °C incubator without CO_2_ for 1 h prior to the assay. OCR measurements were performed using a Seahorse Biosciences XF^e^ Analyzer (Agilent, CA, USA); all experiments were performed at 37 °C. After the measurement of basal respiration, 2 µM oligomycin, and 1 µM carbonyl cyanide-p-trifluoromethoxy phenylhydrazone (FCCP), 1 µM rotenone and antimycin A were added sequentially to measure ATP production, maximal respiratory, and non-mitochondrial respiration, respectively.

The extracellular acidification rate was determined by monitoring the glycolytic function and expressed in mpH/min. The measurement procedure was similar to that of OCR, as described above. After measurement of basal ECAR, 80 mM glucose, 5.0 mM oligomycin, and 100 mM 2-deoxyglucose (2-DG) were added sequentially to determine glycolysis, glycolytic capacity, and glycolytic reserve, respectively.

### Transmission electron microscopy

SW480 and LOVO cells were treated with KS-20073 and KS-20226 for 72 h. The cells were then harvested and fixed with 2% paraformaldehyde (Sigma, K34009105-451) and 2% glutaraldehyde (ZC814139-734). Subsequently, the samples were sliced into 100 nm ultra-thin sections using a microtome. The samples were observed using a transmission electron microscope (JEOL, JEO-1011) in three different fields of view.

### RNA-sequencing and GSEA

The total RNA from control and KS-20,073-treated SW480 and LOVO cells (two biological replicates, *n* = 2) were used for RNA-Seq. GSEA was conducted using the GSEA desktop application (borad.mit.edu/gsea). All genes were ranked using scores based on fold-change direction and p-value, and enrichment analysis was conducted using enrichment static (q-value < 0.5 as a cutoff).

### TCGA data analysis

RNA-seq data from TCGA were obtained from cBioPortal for Cancer Genomics. The log_2_ (RPKM + 1) values of each gene were obtained and analyzed. Top 100 genes showing a positive or negative correlation with *IREB2* expression were selected to obtain the heat map. Gene ontology analysis for biological process was performed with top 100 genes positively correlated with *IREB2* expression. Log-rank maximized test was applied to define optimal cutoff value for categorizing patients according to *IREB2* expression. Overall survival curve was estimated with the Kaplan–Meier method and compared using the log-rank test.

## Electronic supplementary material

Below is the link to the electronic supplementary material.

Supplementary Material 1

## Data Availability

No datasets were generated or analysed during the current study.

## References

[1] Wang J, Chen G, Muckenthaler M, Galy B, Hentze MW, Pantopoulos K. Iron-mediated degradation of IRP2, an unexpected pathway involving a 2-oxoglutarate-dependent oxygenase activity. Mol Cell Biol. 2004;24(3):954–65. 10.1128/mcb.24.3.954-965.2004. 14729944 10.1128/MCB.24.3.954-965.2004 PMC321427

[2] Deng Z, Manz DH, Torti SV, Torti FMJO. Iron-responsive element-binding protein 2 plays an essential role in regulating prostate cancer cell growth. 2017;8(47):82231. 10.18632/oncotarget.19288 PMC5669885 29137259

[3] Wang W, Deng Z, Hatcher H, Miller LD, Di X, Tesfay L, et al. IRP2 regulates breast tumor growth. Cancer Res. 2014;74(2):497–507. 10.1158/0008-5472.CAN-13-1224. 24285726 10.1158/0008-5472.CAN-13-1224 PMC3989290

[4] Anderson CP, Shen M, Eisenstein RS, Leibold EA. Mammalian iron metabolism and its control by iron regulatory proteins. Biochim Biophys Acta. 2012;1823(9):1468–83. 10.1016/j.bbamcr.2012.05.010. 22610083 10.1016/j.bbamcr.2012.05.010 PMC3675657

[5] Bayeva M, Khechaduri A, Puig S, Chang HC, Patial S, Blackshear PJ, et al. mTOR regulates cellular iron homeostasis through tristetraprolin. Cell Metab. 2012;16(5):645–57. 10.1016/j.cmet.2012.10.001. 23102618 10.1016/j.cmet.2012.10.001 PMC3594686

[6] Zhang J, Kong X, Zhang Y, Sun W, Xu E, Chen X. Mdm2 is a target and mediator of IRP2 in cell growth control. FASEB J. 2020;34(2):2301–11. 10.1096/fj.201902278RR. 31907996 10.1096/fj.201902278RR PMC7018553

[7] Miyazawa M, Bogdan AR, Tsuji Y. Perturbation of Iron Metabolism by Cisplatin through Inhibition of Iron Regulatory Protein 2. Cell Chem Biol. 2019;26(1):85–e974. 10.1016/j.chembiol.2018.10.009. 30449675 10.1016/j.chembiol.2018.10.009 PMC6338505

[8] Wang H, Shi H, Rajan M, Canarie ER, Hong S, Simoneschi D, et al. FBXL5 regulates IRP2 Stability in Iron Homeostasis via an oxygen-responsive [2Fe2S] cluster. Mol Cell. 2020. 10.1016/j.molcel.2020.02.011. 32126207 10.1016/j.molcel.2020.02.011 PMC7159994

[9] Jiao Q, Du X, Wei J, Li Y, Jiang H. Oxidative Stress Regulated Iron Regulatory Protein IRP2 through FBXL5-Mediated ubiquitination-proteasome way in SH-SY5Y cells. Front Neurosci. 2019;13:20. 10.3389/fnins.2019.00020. 30760976 10.3389/fnins.2019.00020 PMC6361836

[10] Martelli A, Schmucker S, Reutenauer L, Mathieu JRR, Peyssonnaux C, Karim Z, et al. Iron regulatory protein 1 sustains mitochondrial iron loading and function in frataxin deficiency. Cell Metab. 2015;21(2):311–23. 10.1016/j.cmet.2015.01.010. 25651183 10.1016/j.cmet.2015.01.010

[11] Li H, Liu Y, Shang L, Cai J, Wu J, Zhang W, et al. Iron regulatory protein 2 modulates the switch from aerobic glycolysis to oxidative phosphorylation in mouse embryonic fibroblasts. Proc Natl Acad Sci U S A. 2019;116(20):9871–6. 10.1073/pnas.1820051116. 31040213 10.1073/pnas.1820051116 PMC6525483

[12] Bellelli R, Federico G, Matte A, Colecchia D, Iolascon A, Chiariello M, et al. NCOA4 Deficiency impairs systemic Iron homeostasis. Cell Rep. 2016;14(3):411–21. 10.1016/j.celrep.2015.12.065. 26776506 10.1016/j.celrep.2015.12.065

[13] Wallander ML, Zumbrennen KB, Rodansky ES, Romney SJ, Leibold EA. Iron-independent phosphorylation of iron regulatory protein 2 regulates ferritin during the cell cycle. J Biol Chem. 2008;283(35):23589–98. 10.1074/jbc.M803005200. 18574241 10.1074/jbc.M803005200 PMC2527096

[14] Moroishi T, Nishiyama M, Takeda Y, Iwai K, Nakayama KI. The FBXL5-IRP2 axis is integral to control of iron metabolism in vivo. Cell Metab. 2011;14(3):339–51. 10.1016/j.cmet.2011.07.011. 21907140 10.1016/j.cmet.2011.07.011

[15] Zumbrennen-Bullough KB, Becker L, Garrett L, Holter SM, Calzada-Wack J, Mossbrugger I, et al. Abnormal brain iron metabolism in Irp2 deficient mice is associated with mild neurological and behavioral impairments. PLoS ONE. 2014;9(6):e98072. 10.1371/journal.pone.0098072. 24896637 10.1371/journal.pone.0098072 PMC4045679

[16] Muto Y, Nishiyama M, Nita A, Moroishi T, Nakayama KI. Essential role of FBXL5-mediated cellular iron homeostasis in maintenance of hematopoietic stem cells. Nat Commun. 2017;8:16114. 10.1038/ncomms16114. 28714470 10.1038/ncomms16114 PMC5520054

[17] Bray F, Ferlay J, Soerjomataram I, Siegel RL, Torre LA, Jemal A. Global cancer statistics 2018: GLOBOCAN estimates of incidence and mortality worldwide for 36 cancers in 185 countries. CA Cancer J Clin. 2018;68(6):394–424. 10.3322/caac.21492. 30207593 10.3322/caac.21492

[18] Missiaglia E, Jacobs B, D’Ario G, Di Narzo AF, Soneson C, Budinska E, et al. Distal and proximal colon cancers differ in terms of molecular, pathological, and clinical features. Ann Oncol. 2014;25(10):1995–2001. 10.1093/annonc/mdu275. 25057166 10.1093/annonc/mdu275

[19] Van Cutsem E, Kohne CH, Lang I, Folprecht G, Nowacki MP, Cascinu S, et al. Cetuximab plus Irinotecan, fluorouracil, and leucovorin as first-line treatment for metastatic colorectal cancer: updated analysis of overall survival according to tumor KRAS and BRAF mutation status. J Clin Oncol. 2011;29(15):2011–9. 10.1200/JCO.2010.33.5091. 21502544 10.1200/JCO.2010.33.5091

[20] Andre T, Shiu KK, Kim TW, Jensen BV, Jensen LH, Punt C, et al. Pembrolizumab in microsatellite-instability-high Advanced Colorectal Cancer. N Engl J Med. 2020;383(23):2207–18. 10.1056/NEJMoa2017699. 33264544 10.1056/NEJMoa2017699

[21] Siegel RL, Miller KD, Jemal A. Cancer statistics, 2019. CA Cancer J Clin. 2019;69(1):7–34. 10.3322/caac.21551. 10.3322/caac.21551 30620402

[22] Sivaprakasam S, Ristic B, Mudaliar N, Hamood AN, Colmer-Hamood J, Wachtel MS, et al. Hereditary hemochromatosis promotes colitis and colon cancer and causes bacterial dysbiosis in mice. Biochem J. 2020;477(19):3867–83. 10.1042/BCJ20200392. 32955078 10.1042/BCJ20200392 PMC7557149

[23] Heath JL, Weiss JM, Lavau CP, Wechsler DS. Iron deprivation in cancer–potential therapeutic implications. Nutrients. 2013;5(8):2836–59. 10.3390/nu5082836. 23887041 10.3390/nu5082836 PMC3775231

[24] Guggisberg CA, Kim J, Lee J, Chen X, Ryu MS. NCOA4 regulates Iron Recycling and responds to Hepcidin Activity and Lipopolysaccharide in macrophages. Antioxid (Basel). 2022;11(10). 10.3390/antiox11101926. 10.3390/antiox11101926 PMC9598790 36290647

[25] Newman SA, Pan Y, Short JL, Nicolazzo JA. Increasing intracellular levels of Iron with Ferric Ammonium Citrate Leads to reduced P-glycoprotein expression in human immortalised brain microvascular endothelial cells. Pharm Res. 2021;38(1):97–111. 10.1007/s11095-021-03006-y. 33532991 10.1007/s11095-021-03006-y

[26] Muzny DM, Bainbridge MN, Chang K, Dinh HH, Drummond JA, Fowler G, et al. Compr Mol Charact Hum colon Rectal cancer. 2012;487(7407):330–7. 10.1038/nature11252 PMC3401966 22810696

[27] Song Y, Kim JS, Kim SH, Park YK, Yu E, Kim KH, et al. Patient-derived multicellular tumor spheroids towards optimized treatment for patients with hepatocellular carcinoma. J Exp Clin Cancer Res. 2018;37(1):109. 10.1186/s13046-018-0752-0. 29801504 10.1186/s13046-018-0752-0 PMC5970513

[28] Hara Y, Yanatori I, Tanaka A, Kishi F, Lemasters JJ, Nishina S, et al. Iron loss triggers mitophagy through induction of mitochondrial ferritin. EMBO Rep. 2020;21(11):e50202. 10.15252/embr.202050202. 32975364 10.15252/embr.202050202 PMC7645172

[29] You L, Wang Z, Li H, Shou J, Jing Z, Xie J, et al. The role of STAT3 in autophagy. Autophagy. 2015;11(5):729–39. 10.1080/15548627.2015.1017192. 25951043 10.1080/15548627.2015.1017192 PMC4509450

[30] Crielaard BJ, Lammers T, Rivella S. Targeting iron metabolism in drug discovery and delivery. Nat Rev Drug Discov. 2017;16(6):400–23. 10.1038/nrd.2016.248. 28154410 10.1038/nrd.2016.248 PMC5455971

[31] Torti SV, Torti FM. Iron and Cancer: 2020 vision. Cancer Res. 2020;80(24):5435–48. 10.1158/0008-5472.Can-20-2017. 10.1158/0008-5472.CAN-20-2017 PMC8118237 32928919

[32] Torti SV, Torti FM. Iron and cancer: more ore to be mined. Nat Rev Cancer. 2013;13(5):342–55. 10.1038/nrc3495. 23594855 10.1038/nrc3495 PMC4036554

[33] Muckenthaler MU, Rivella S, Hentze MW, Galy B. A Red Carpet for Iron Metabolism. Cell. 2017;168(3):344–61. 10.1016/j.cell.2016.12.034. 28129536 10.1016/j.cell.2016.12.034 PMC5706455

[34] Martin MG, Arcasoy MO. Deferasirox versus deferoxamine. Blood. 2006;108(2):774–5. 10.1182/blood-2006-02-002436. author reply 5–6. 16822908 10.1182/blood-2006-02-002436

[35] Seebacher NA, Richardson DR, Jansson PJ. A mechanism for overcoming P-glycoprotein-mediated drug resistance: novel combination therapy that releases stored doxorubicin from lysosomes via lysosomal permeabilization using Dp44mT or DpC. Cell Death Dis. 2016;7(12):e2510. 10.1038/cddis.2016.381. 27906178 10.1038/cddis.2016.381 PMC5261000

[36] Dixon SJ, Stockwell BR. The role of iron and reactive oxygen species in cell death. Nat Chem Biol. 2014;10(1):9–17. 10.1038/nchembio.1416. 24346035 10.1038/nchembio.1416

[37] Nunnari J, Suomalainen A. Mitochondria: in sickness and in health. Cell. 2012;148(6):1145–59. 10.1016/j.cell.2012.02.035. 22424226 10.1016/j.cell.2012.02.035 PMC5381524

[38] Letts JA, Sazanov LA. Clarifying the supercomplex: the higher-order organization of the mitochondrial electron transport chain. Nat Struct Mol Biol. 2017;24(10):800–8. 10.1038/nsmb.3460. 28981073 10.1038/nsmb.3460

[39] Puy H, Gouya L, Deybach JC, Porphyrias. Lancet. 2010;375(9718):924–37. 10.1016/s0140-6736(09)61925-5. 20226990 10.1016/S0140-6736(09)61925-5

[40] Brissot P, Pietrangelo A, Adams PC, de Graaff B, McLaren CE, Loréal O, Haemochromatosis. Nat Rev Dis Primers. 2018;4:18016. 10.1038/nrdp.2018.16. 29620054 10.1038/nrdp.2018.16 PMC7775623

[41] Fleming RE, Ponka P. Iron overload in human disease. N Engl J Med. 2012;366(4):348–59. 10.1056/NEJMra1004967. 22276824 10.1056/NEJMra1004967

[42] Hassannia B, Vandenabeele P, Vanden Berghe T. Targeting ferroptosis to Iron Out Cancer. Cancer Cell. 2019;35(6):830–49. 10.1016/j.ccell.2019.04.002. 31105042 10.1016/j.ccell.2019.04.002

[43] Kimmelman AC, White E. Autophagy and Tumor Metabolism. Cell Metab. 2017;25(5):1037–43. 10.1016/j.cmet.2017.04.004. 28467923 10.1016/j.cmet.2017.04.004 PMC5604466

[44] Cloonan SM, Glass K, Laucho-Contreras ME, Bhashyam AR, Cervo M, Pabon MA, et al. Mitochondrial iron chelation ameliorates cigarette smoke-induced bronchitis and emphysema in mice. Nat Med. 2016;22(2):163–74. 10.1038/nm.4021. 26752519 10.1038/nm.4021 PMC4742374

