# Correction: Inhibition of IRP2-dependent reprogramming of iron metabolism suppresses tumor growth in colorectal cancer

**DOI:** 10.1186/s12964-024-01840-2

**Published:** 2024-10-07

**Authors:** Jieon Hwang, Areum Park, Chinwoo Kim, Chang Gon Kim, Jaesung Kwak, Byungil Kim, Hyunjin Shin, Minhee Ku, Jaemoon Yang, Ayoung Baek, Jiwon Choi, Hocheol Lim, Kyoung Tai No, Xianghua Zhao, Uyeong Choi, Tae Il Kim, Kyu-Sung Jeong, Hyuk Lee, Sang Joon Shin

**Affiliations:** 1 Department of Medicine, Yonsei University College of Medicine, Seoul, 03722 Korea; 2 Songdang Institute for Cancer Research, Yonsei University College of Medicine, Seoul, 03722 Korea; 3 Division of Medical Oncology, Department of Internal Medicine, Yonsei Cancer Center, Yonsei University College of Medicine, Seoul, 03722 Korea; 4 Infectious Diseases Therapeutic Research Center, Korea Research Institute of Chemical Technology, Daejeon, 34114 Korea; 5 Department of Chemistry, Yonsei University, Seoul, 03722 Korea; 6 Division of Gastroenterology, Department of Internal Medicine, Yonsei University College of Medicine, Seoul, 03722 Korea; 7 Department of Radiology, Yonsei University College of Medicine, Seoul, 03722 Korea; 8 Convergence Research Center for Systems Molecular Radiological Science, Yonsei University, Seoul, 03722 Korea; 9 Bioinformatics and Molecular Design Research Center, Incheon, 21983 Korea; 10 The Interdisciplinary Graduate Program in Integrative Biotechnology & Translational Medicine, Yonsei University, Incheon, 21983 Korea; 11 College of Pharmacy, Dongduk Women’s University, Seoul, 02748 Korea

**Correction: Cell Commun Signal 22, 412 (2024)**

10.1186/s12964-024-01769-6

Following the publication of the original article [1], the authors noticed in error in the funding section. Grant number KK2032-10 should be replaced with KK2032-20. The incorrect and correct funding note is given below:

Incorrect: This research was supported by the National Research Foundation of Korea (NRF) grant funded by the Korea government (MSIT) (2022R1A2C2004738), the Korea Research Institute of Chemical Technology (Grant No. SI-1404, KK2332-10, KK2032-10) and by the Quantum Advantage challenge research based on Quantum Computing through the National Research Foundation of Korea (NRF) funded by the Ministry of Science and ICT (RS-2023-00257288).

Correct: This research was supported by the National Research Foundation of Korea (NRF) grant funded by the Korea government (MSIT) (2022R1A2C2004738), the Korea Research Institute of Chemical Technology (Grant No. SI-1404, KK2332-10, KK2032-20) and by the Quantum Advantage challenge research based on Quantum Computing through the National Research Foundation of Korea (NRF) funded by the Ministry of Science and ICT (RS-2023-00257288).

The original article [1] has been corrected.
