# Supplementary material for: Inhibition of IRP2-dependent reprogramming of iron metabolism suppresses tumor growth in colorectal cancer
Source: Cell Commun Signal. 2024 Aug 23;22:412. doi: 10.1186/s12964-024-01769-6 (PMC11342626; doi:10.1186/s12964-024-01769-6)
Supplement: Supplementary file 1 — Supplementary Material 1 [file 12964_2024_1769_MOESM1_ESM.docx]

**Supplemental Data File**

**Inhibition of IRP2-dependent reprogramming of iron metabolism suppresses tumor growth in colorectal cancer**

Jieon Hwang^1,2†^, Areum Park^4,5†^, Chinwoo Kim^1,2^, Chang Gon Kim^3^, Jaesung Kwak^4^, Byungil Kim^4^, Hyunjin Shin^4^, Minhee Ku^7,8^, Jaemoon Yang^7,8^, Ayoung Baek^9^, Jiwon Choi^11^, Hocheol Lim^9^, Kyoung Tai No^9,10^, Xianghua Zhao^2^, Uyeong Choi^1,2^, Tae Il Kim^6^, Kyu-Sung Jeong^5^, Hyuk Lee^4*^ and Sang Joon Shin^2,3,8,13*^

**Supplemental Figures**

**
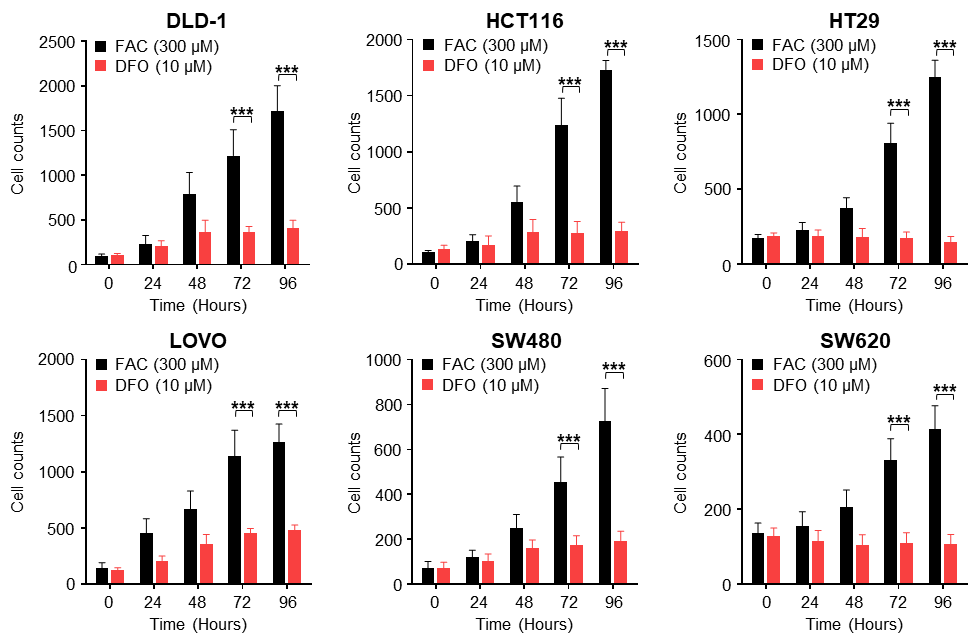
**

**Supplemental Figure S1. Iron regulates cell growth through the disruption of iron metabolism** (Related to Figure 1).

Effect of ferric ammonium citrate (FAC) (300 µM) and DFO (10 µM) on growth in CRC cell lines. Cells were monitored every 24 hours and 8 random fields were counted using the Harmony software. Data are represented as the mean ± SEM are shown (n = 8). ***p < 0.005.

**
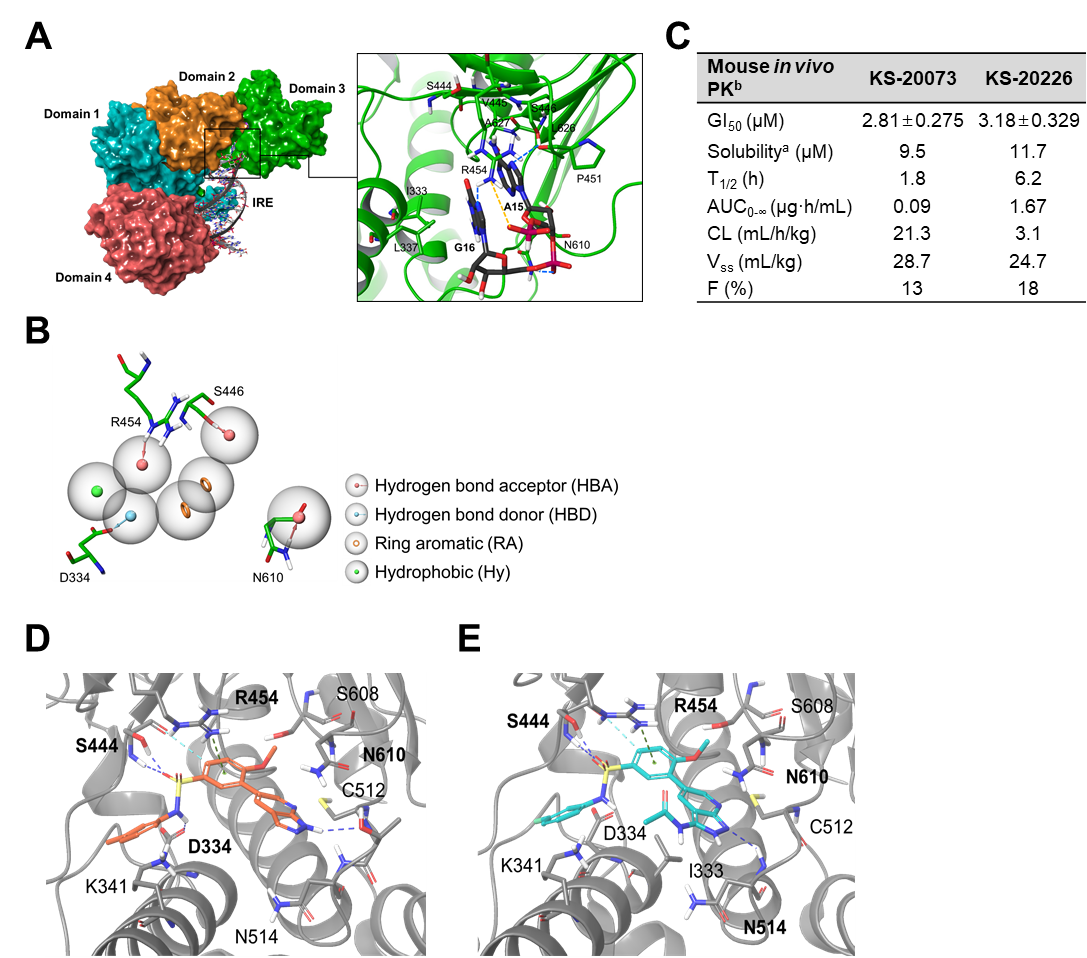
**

**Supplemental Figure S2. Homology modeling of the IRP2-IRE complex and characterization of IRP2 inhibitors** (Related to Figure 3).

**A.** The predicted 3D structure of IRP2 is shown as surface and the IRE is represented by ribbon (left panel). IRP2 proteins are shown using ribbon representation. The key interacting residues on IRP2 are represented using green stick models. A15 and G16 are represented using a gray stick. The hydrogen bond interaction is depicted by a blue dashed line, and the salt bridge interaction is represented by a yellow dashed line (right panel). **B.** The final pharmacophore model comprised three hydrogen bond acceptors (HBA), one hydrogen bond donor (HBD), one ring aromatic (RA), and one hydrophobic (Hy) feature. The key residues are represented using the stick model. c Characteristic of KS-20073 and KS-20226. Acid dissociation constant (PK_a_) and equilibrium solubility of KS-20073 and KS-20226. ^a^Solubility of compounds in aqueous phosphate buffer at pH 7.4 after 16 h at 25 °C from DMSO stock solution. ^b^KS-20073 was dosed at 2 mg/kg I.V and 10 mg/kg P.O KS-20226 was dosed at 5 mg/kg I.V and 10 mg/kg P.O. **C, D.** Mode of KS-20073 **C.** and KS-20226 **D.** binding on IRP2. KS-20073 and KS-20226 dock at the IRE terminal-loop binding pocket. IRP2 protein is depicted using ribbon representation. The key interacting residues are represented using a gray stick. The hydrogen bond interaction is represented using a blue dashed line, and π-cation interactions are depicted by a green dashed line.

**
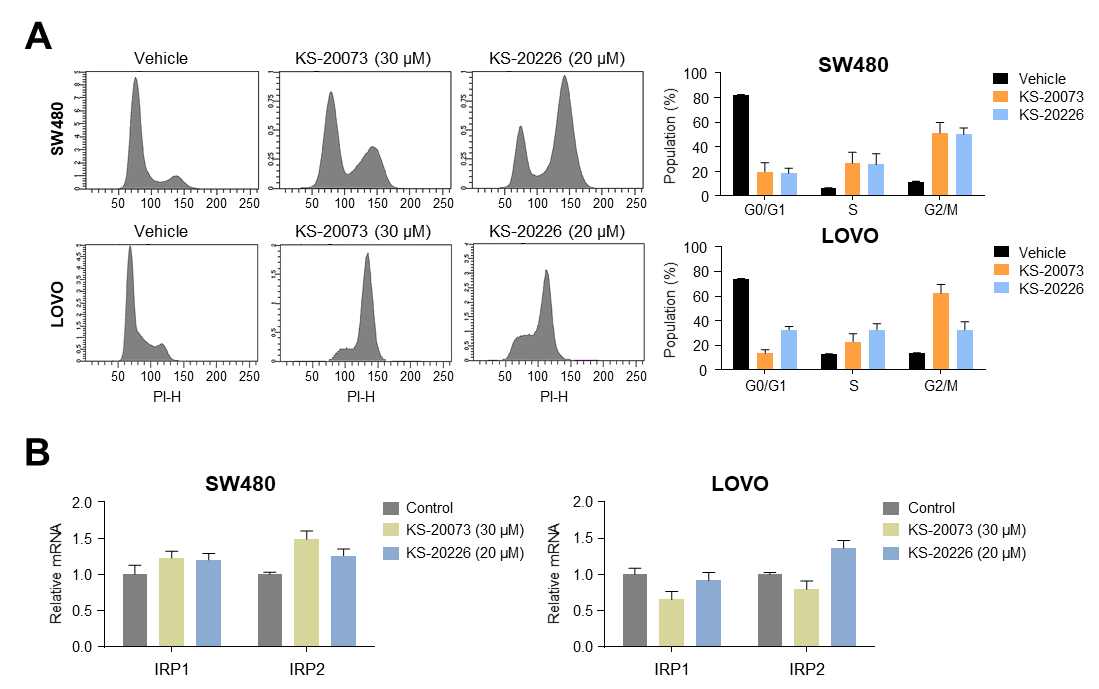
**

**Supplemental Figure S3. IRP2 inhibition induces cell cycle arrest and has a slight effect on transcriptional activity** (Related to Figure 4).

**A.** The cell cycle of IRP2 inhibition was investigated using flow cytometry. Data are represented as the mean ± SEM (n = 3). **B.** IRP1 and IRP2 mRNA levels measured using qRT-PCR.


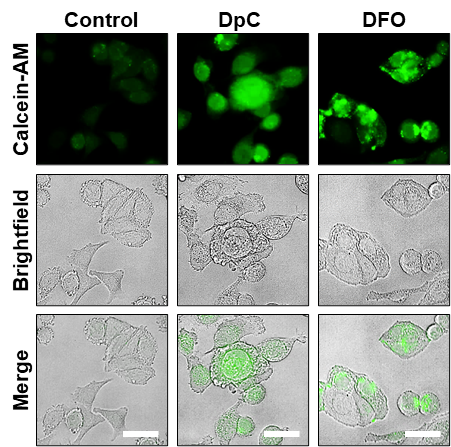


**Supplemental Figure S4. Effect of deferoxamine (DFO) and DpC on the labile iron pool (LIP)** (Related to Figure 4).

Labile iron pool (LIP) was determined using Calcein AM (green). SW480 cell was treated with DFO (100 µM) and DpC (5 µM), ion chelating agents, as the positive control.

**
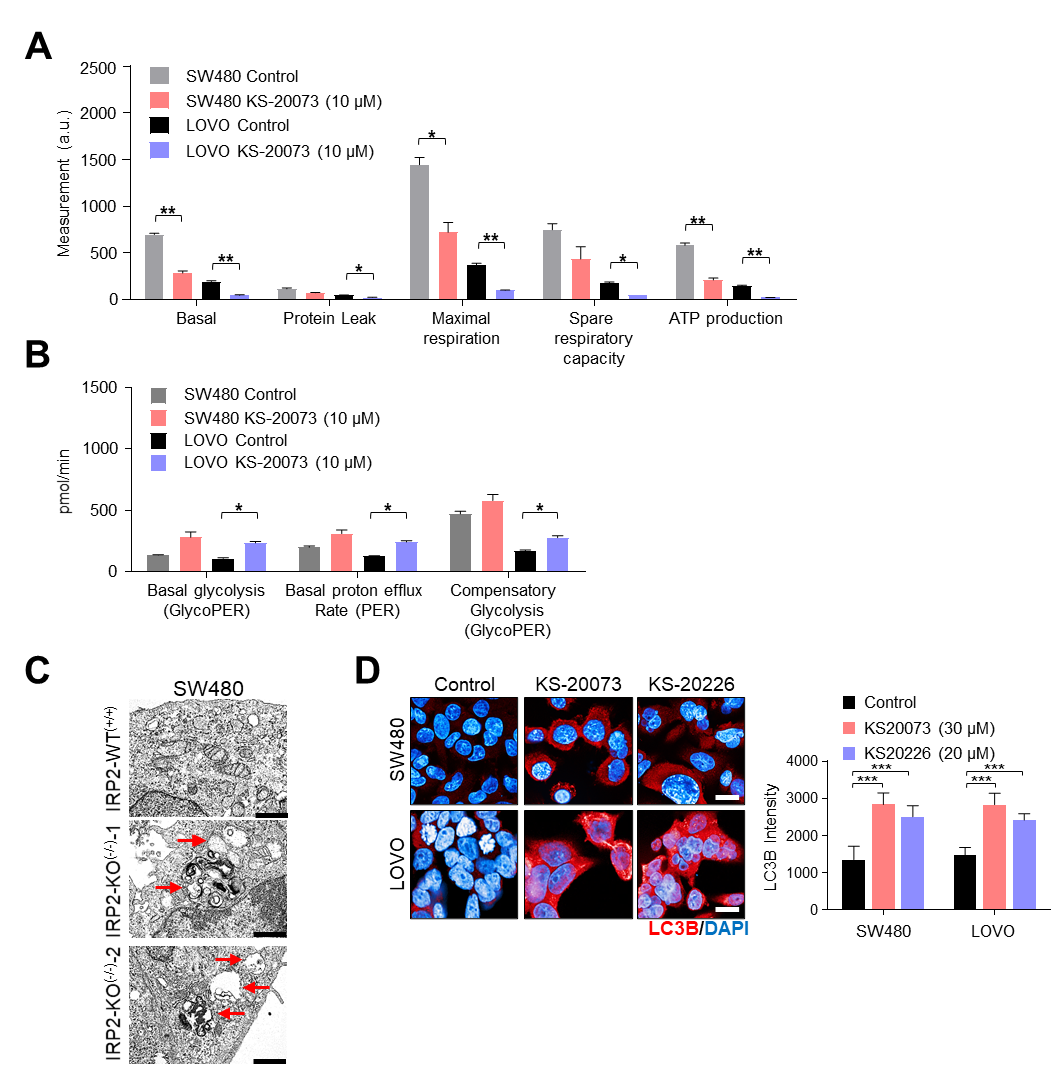
**

**Supplemental Figure S5. IRP2 inhibition causes mitochondrial damage and dominates autophagy** (Related to Figure 5).

**A, B.** Mitochondrial maximal respiration, spare respiratory capacity, ATP production **A**, basal glycolysis, and compensatory glycolysis level **B** of KS20073 were calculated using XF cell energy phenotype test. Data are represented as the mean ± SEM (n = 3). **C.** Representative transmission electron microscopy (TEM) images of IRP2 knockout SW480 cell for 48 hours. The red arrowheads indicate the autophagosomes. Scale bars in figures are 2000 nm. **D.** Representative immunofluorescence images of LC3B and DAPI (blue) in SW480 and LOVO cells treated with KS-20073 (30 µM) and KS-20226 (20 µM) for 48 hours were obtained using confocal microscopy. The fluorescence intensity was measured and quantified using the Harmony software. Data are represented as the mean ± SEM (n = 4). ***p < 0.005.


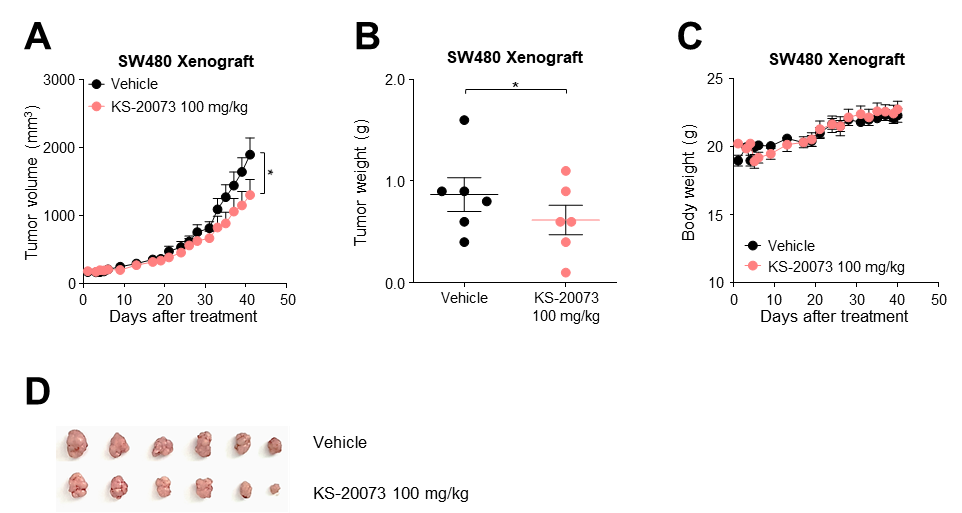


**Supplemental Figure S6. Inhibition of IRP2 suppressed tumor growth in xenograft model.** (Related to Figure 6).

**A.** Tumor volumes of SW480 xenografts injected intraperitoneally with vehicle or KS-20073 (100 mg/kg). Data are represented as the mean ± SEM (n = 6). *p < 0.05. **B.** Tumor weights of SW480 xenografts with vehicle or KS-20073 (100 mg/kg). Data are represented as the mean ± SEM (n = 6). *p < 0.05. **C.** Body weight after treatment of KS-20073 (100 mg/kg) for 44 days. **D.** Representative tumor images in SW480 xenografts with vehicle and KS-20073 (100 mg/kg).

**Supplemental Table S1. The characteristics of tissue derived from individual CRC patients.** ICDO; Stage; EGFR, BRAF, and KRAS mutation status; and MSS/MSI-H status are shown.

*ICDO: International Classification of Diseases for Oncology

|  | **Y-005** | **Y-011** | **Y-012** | **Y-015** |
| --- | --- | --- | --- | --- |
| ICDO* | Rectosigmoid junction | Rectum, NOS | Ascending Colon | Rectum, NOS |
| EGFR | None | None | None | None |
| BRAF | None | None | None | None |
| KRAS | Wild | Wild | Wild | Wild |
| Microsatellite  instability | MSS | MSS | MSS | MSS |

|  | **Y-016** | **Y-035** | **Y-040** | **Y-041** | **Y-047** |
| --- | --- | --- | --- | --- | --- |
| 2ICDO* | Sigmoid  Colon | Sigmoid Colon | Ascending Colon | Transverse Colon | Rectosigmoid junction |
| EGFR | None | None | None | None | Focal and moderate staining |
| BRAF | None | None | None | None | None |
| KRAS | G12V | Wild | G12V | Wild | Wild |
| Microsatellite  instability | MSS | MSS | MSS | MSI-H | MSS |

**Supplemental Methods**

**Homology modeling**

We performed homology modeling to predict the 3D structure of the human IRP2–IRE complex using Small-molecule Drug Discovery Suite 2018-3 [54, 55]. The full sequence of the human IRP2 was retrieved from the UniProt database (Accession No: P48200), and the X-ray crystal structure of human IRP1 including IRE (PEB ID: 3SNP, resolution: 2.8 Å), and Cryo-EM structure of human IRP2 (PDB ID: 6VCD, resolution: 3.0 Å) were used as template structures. Protein Preparation Wizard was used to assign bond orders, charge states to ionizable residues, and perform a restrained minimization of the IRP2–IRE complex. Hydrogen atoms were added to the model structure at pH 7.0, and their positions were optimized with the PROPKA function in the Maestro program.

**Pharmacophore model development and virtual screening**

The pharmacophore model was generated from the IRP2–IRE complex using the PHASE module of Maestro [1, 2]. It consisted of seven features: three HBA, one HBD, one Hy, and two RA. To improve the effectiveness of virtual screening, the excluded volumes were added to the pharmacophore model. Structure-based virtual screening was performed using an in-house database that included synthetic compounds and natural products (approximately 7.0 million compounds). The hit compounds were screened using the pharmacophore model. The final virtual hits were selected by fitness score > 1.0 and visual inspection.

**Molecular docking simulation**

The 2D structures of the hit compounds were converted to the geometrically refined 3D structures using the LigPrep module in Maestro [3-5]. All possible ionization and protonation states, tautomers, stereochemistry, and ring conformations were generated and assigned proper bond orders. Next, the grid box was generated using a receptor grid generation module, and set at a distance of 10 Å from the critical residue on the IRE terminal-loop binding pocket. The Glide extra-precision (XP) docking was conducted to determine the binding mode of hit compounds on IRP2 using Maestro [6-8].

**Fragment molecular orbital (FMO) calculation**

All FMO calculations were performed with the version dated Feb 14, 2018, GAMESS. The two-body FMO method was applied to IRP2–IRE complex and three IRP2-ligand complexes at the second order Møller-Plesset perturbation theory and polarizable continuum model with 6-31G** basis set (FMO2-MP2/6-31G**/PCM level). The fragmentation was in compliance with the hybrid orbital projection scheme, where the fragmentation is performed at the *sp*3 bond between two carbons, not a peptide bond in protein and not a phosphodiester bond in RNA. The fragmentation was done at the bond between alpha carbon atom and carbonyl carbon atom in the protein backbone and at the bond between 5′ carbon atom and 4′ carbon atom in the RNA backbone. Only the protein residues within 10.4 Å from RNA in the IRP2–IRE complex or from ligand in IRP2–ligand complex were included in each FMO calculation. The significant interactions in IRP2–IRE complex were selected with 3D-SPIEs method, which considered a pair interaction to be significant if stability > -3.0 kcal/mol and the interaction occurred within 5.4 Å.

**References**

1. Dixon SL, Smondyrev AM, Rao SN. PHASE: a novel approach to pharmacophore modeling and 3D database searching. Chem Biol Drug Des. 2006;67(5):370-2.

2. Dixon SL, Smondyrev AM, Knoll EH, Rao SN, Shaw DE, Friesner RA. PHASE: a new engine for pharmacophore perception, 3D QSAR model development, and 3D database screening: 1. Methodology and preliminary results. J Comput Aided Mol Des. 2006;20(10-11):647-71.

3. Sastry GM, Adzhigirey M, Day T, Annabhimoju R, Sherman W. Protein and ligand preparation: parameters, protocols, and influence on virtual screening enrichments. J Comput Aided Mol Des. 2013;27(3):221-34.

4. Shelley JC, Cholleti A, Frye LL, Greenwood JR, Timlin MR, Uchimaya M. Epik: a software program for pK( a ) prediction and protonation state generation for drug-like molecules. J Comput Aided Mol Des. 2007;21(12):681-91.

5. Greenwood JR, Calkins D, Sullivan AP, Shelley JC. Towards the comprehensive, rapid, and accurate prediction of the favorable tautomeric states of drug-like molecules in aqueous solution. J Comput Aided Mol Des. 2010;24(6-7):591-604.

6. Friesner RA, Murphy RB, Repasky MP, Frye L, Greenwood JR, Halgren TA, et al. Extra precision glide: docking and scoring incorporating a model of hydrophobic enclosure for protein-ligand complexes. Journal of Medicinal Chemistry (formerly: Journal of Medicinal and Pharmaceutical Chemistry). 2006;49(21):6177-96.

7. Friesner RA, Banks JL, Murphy RB, Halgren TA, Klicic J, Mainz DT, et al. Glide: a new approach for rapid, accurate docking and scoring. 1. Method and assessment of docking accuracy. Journal of Medicinal Chemistry (formerly: Journal of Medicinal and Pharmaceutical Chemistry). 2004;47(7):1739-49.

8. Halgren TA, Murphy RB, Friesner RA, Beard HS, Frye L, Pollard WT, et al. Glide: a new approach for rapid, accurate docking and scoring. 2. Enrichment factors in database screening. Journal of Medicinal Chemistry (formerly: Journal of Medicinal and Pharmaceutical Chemistry). 2004;47(7):1750-9.
